# STING‐dependent induction of neutrophilic asthma exacerbation in response to house dust mite

**DOI:** 10.1111/all.16369

**Published:** 2024-10-28

**Authors:** Yasmine Messaoud‐Nacer, Elodie Culerier, Stéphanie Rose, Isabelle Maillet, Rania Boussad, Chloé Veront, Florence Savigny, Bernard Malissen, Urszula Radzikowska, Milena Sokolowska, Gabriel V. L. da Silva, Michael R. Edwards, David J. Jackson, Sebastian L. Johnston, Bernhard Ryffel, Valerie F. Quesniaux, Dieudonnée Togbe

**Affiliations:** ^1^ Experimental and Molecular Immunology and Neurogenetics INEM UMR7355 University of Orleans and CNRS Orleans France; ^2^ Centre d'Immunophénomique (CIPHE) Aix Marseille Université, INSERM, CNRS Marseille France; ^3^ Swiss Institute of Allergy and Asthma Research (SIAF) University of Zurich Davos Switzerland; ^4^ Christine Kühne—Center for Allergy Research and Education (CK‐CARE) Herman‐ Burchard‐Strasse 1 Davos Switzerland; ^5^ Ribeirão Preto Medical School University of Sao Paulo Sao Paulo Brazil; ^6^ National Heart and Lung Institute Imperial College Londont London UK; ^7^ Asthma UK Centre in Allergic Mechanism of Asthma London UK; ^8^ Imperial College Healthcare NHS Trust London UK; ^9^ Guy’s Severe Asthma Centre, Guy’s & St Thomas’ NHS Trust London UK; ^10^ School of Immunology & Microbial Sciences, King’s College London London UK

**Keywords:** asthma exacerbation, cell death, cGAMP, diABZI, DNA sensing, with inserts

## Abstract

**Background:**

Severe refractory, neutrophilic asthma remains an unsolved clinical problem. STING agonists induce a neutrophilic response in the airways, suggesting that STING activation may contribute to the triggering of neutrophilic exacerbations. We aim to determine whether STING‐induced neutrophilic lung inflammation mimics severe asthma.

**Methods:**

We developed new models of neutrophilic lung inflammation induced by house dust mite (HDM) plus STING agonists diamidobenzimidazole (diABZI) or cGAMP in wild‐type, and conditional‐STING‐deficient mice. We measured DNA damage, cell death, NETs, cGAS/STING pathway activation by immunoblots, N1/N2 balance by flow cytometry, lung function by plethysmography, and Th1/Th2 cytokines by multiplex. We evaluated diABZI effects on human airway epithelial cells from healthy or patients with asthma, and validated the results by transcriptomic analyses of rhinovirus infected healthy controls vs patients with asthma.

**Results:**

DiABZI administration during HDM challenge increased airway hyperresponsiveness, neutrophil recruitment with prominent NOS2^+^ARG1^−^ type 1 neutrophils, protein extravasation, cell death by PANoptosis, NETs formation, extracellular dsDNA release, DNA sensors activation, IFNγ, IL‐6 and CXCL10 release. Functionally, STING agonists exacerbated airway hyperresponsiveness. DiABZI caused DNA and epithelial barrier damage, STING pathway activation in human airway epithelial cells exposed to HDM, in line with DNA‐sensing and PANoptosis pathways upregulation and tight‐junction downregulation induced by rhinovirus challenge in patients with asthma.

**Conclusions:**

Our study identifies that triggering STING in the context of asthma induces cell death by PANoptosis, fueling the flame of inflammation through a mixed Th1/Th2 immune response recapitulating the features of severe asthma with a prognostic signature of type 1 neutrophils.

## Graphical abstract

This study investigates whether STING‐induced neutrophilic lung inflammation mimics severe asthma. STING activation during allergic challenge triggers neutrophilic asthma accompanied by a Th1 and pro‐inflammatory cytokine in addition to the Th2 cytokines resulting in an epithelial barrier damage, cell death and self‐dsDNA release with increased mucus hypersecretion. STING agonists induce N2 (ARG1^+^ NOS2^‐^) versus N1 (NOS2^+^ ARG1^‐^) neutrophil polarization and NETs formation. ARG1, arginase 1; cGAMP, cyclic guanosine monophosphate–adenosine monophosphate; cGAS, cyclic GMP‐AMP synthase; diABZI, diamidobenzimidazole; EOS, eosinophil; HDM, house dust mite; IL, interleukin; MΦ, macrophage; MO, monocyte; MPO, myeloperoxydase; NEU, neutrophil; NOS2, nitric oxide synthase 2; STING, stimulator of interferon genes; Th, T helper cell

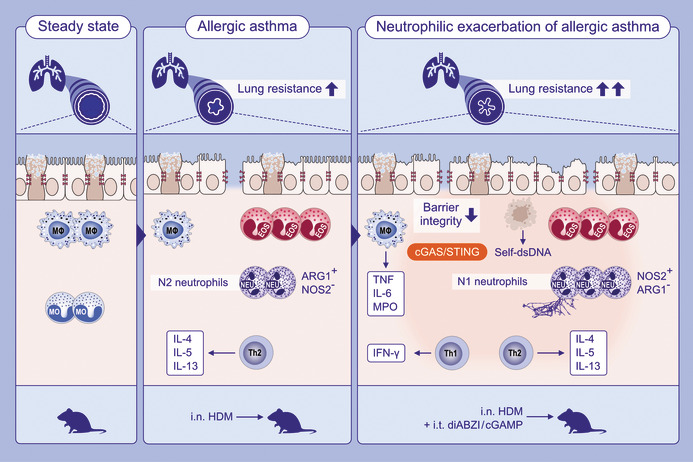

**Abbreviations**
AHR: airway hyperresponsiveness
AIM2: absent in melanoma 2
BAL: bronchoalveolar lavage
BALF: bronchoalveolar lavage fluid
cGAMP: cyclic guanosine monophosphate‐adenosine monophosphate
cGAS: cyclic GMP‐AMP synthase
DDX41: DEAD‐box helicase 41
diABZI: symmetry‐related amidobenzimidazole‐based compounds
GEO: gene expression omnibus
GSDMD: gasdermin D
hAEC: human airway epithelial cells
HBEC: human bronchial epithelial cells
HDM: house dust mite
HE: hematoxylin and eosin
IFI204: IFN‐γ‐Inducible protein 204
IRF3: IFN regulatory factor 3
ISG: interferon stimulated gene
LDH: lactate dehydrogenase
MLKL: mixed lineage kinase domain likepseudokinase
MPO: myeloperoxidase
NETs: neutrophil extracellular traps
NLRP3: NOD‐ LRR‐ and Pyrin Domain‐Containing Protein 3
PAS: periodic acid Schiff
RNA‐Seq: RNA sequencing
STING: stimulator of interferon genes
TBK1: TANK‐binding kinase 1
WT: wild type
ZBP1: Z‐DNA‐Binding Protein 1

## 1 INTRODUCTION

The most common reaction to several allergens such as house‐dust mite (HDM) in allergic asthma endotype is IgE production depending on high Th2 responses with IL‐4, IL‐5 and IL‐13 release, mucus overproduction, eosinophil recruitment, epithelial metaplasia and airway remodeling.^1^ The ability of an allergen to initiate the first steps of type 2 immune response is based on direct epithelial cell damage and damage of associated cells forming the respiratory barrier. Allergenic toxins or proteases may cause necrotic cell death leading to a strong Th2 response.^2^ Severe asthma is often associated with Th2 low and mixed endotype, increased neutrophils and extracellular DNA (eDNA) in patient sputum, with an increase in type 1 cytokines IL‐1β and IL‐6 at the late onset of the disease, and unresponsiveness to corticosteroids or specific biologicals.^1^, ^3^, ^4^, ^5^, ^6^ However, the molecular mechanism underlying severe neutrophilic asthma is more complex and remains unsolved.

Extracellular host‐derived DNA has been associated with allergic type 2 immune responses. Indeed, host DNA released from dying cells acts as a damage associated molecular pattern (DAMP) that mediates for example, the adjuvant activity of alum.^7^ The detection of extracellular host‐derived dsDNA is ensured by cyclic GMP–AMP synthase (cGAS) which converts ATP and GTP into cyclic dinucleotide 2'3'cGAMP. The latter, in turn binds and activates the ER‐resident adaptor protein Stimulator of Interferon Genes (STING) which activates the transcription factors NF‐kB and IRF3, and the production of pro‐inflammatory cytokines including tumor necrosis factor (TNFα), IL‐6 and type I IFNs (IFNα/β).^8^

Cytosolic dsDNA accumulated in airway epithelia of ovalbumin (OVA)‐ or HDM‐challenged mice.^9^ Deletion of cGAS in airway epithelial cells attenuated OVA or HDM induced allergic airway inflammation, while cGAS promoted Th2 immunity likely by regulating airway epithelial GM‐CSF^9^. Further, 2'3'cGAMP or c‐di‐GMP produced after activation of cGAS by extracellular DNA, was shown to function as an adjuvant promoting type 2 allergic lung inflammation when administered during sensitization with HDM, with increased of serum IgE and eosinophils in the airways, an effect mediated by IL‐33 and STING signaling pathway.^10^, ^11^

Recently, we showed that endotracheal administration of cGAMP or the synthetic STING agonist diamidobenzimidazole (diABZI) led to lung inflammation with neutrophilic response in the broncho‐alveolar space, cell death via PANoptosis, loss of epithelial barrier function, and release of self‐dsDNA and NETs.^12^

Here, we asked whether activating STING pathway during challenges after immunization with the widely distributed house‐dust mite allergen might affect the allergic response and trigger a neutrophilic exacerbation. We show that cGAMP and furthermore diABZI administration during challenge in mice exacerbated HDM‐induced lung resistance, cell death by PANoptosis, neutrophil recruitment with a prominent N2 to N1 switch, protein extravasation and extracellular dsDNA release in the airways, with an overexpression of Th1 cytokine IFNγ. We report differential expression of DNA‐sensing and PANoptosis pathways after rhinovirus challenge in patients with asthma.

## 2 METHODS

### 2.1 Mice

Female C57BL/6Rj mice were purchased from Janvier Laboratories (Le Genest St Isle, France). Wild type mice and mice deficient for STING (STING^−/−^)^13^ or cGAS (cGAS^−/−^)^14^ were bred and housed under specific pathogen free conditions at CNRS animal facility (TAAM UAR44, Orleans, France). STING‐OST^fl^ were crossed to LysM^Cre^ mice to generate STING‐OST^fl^ LysM^cre^ mice.^15^ They were maintained in a 12‐h light–dark cycle with food and water ad libitum, following European and local legislation. Age‐matched, 8‐ to 12‐ week‐old mice were used for experiments. All animal experiments complied with the French Government animal experiment regulations and ARRIVE guidelines. The protocols were submitted to the “Ethics Committee for Animal Experimentation of CNRS Campus Orleans” under the number CLE CCO 2019–2017 and 2020–2006, and approved by the French Minister under APAFIS #25876 and #26195.

### 2.2 Induction of allergic airway inflammation and bronchoalveolar lavage (BAL)

**FIGURE 1:**
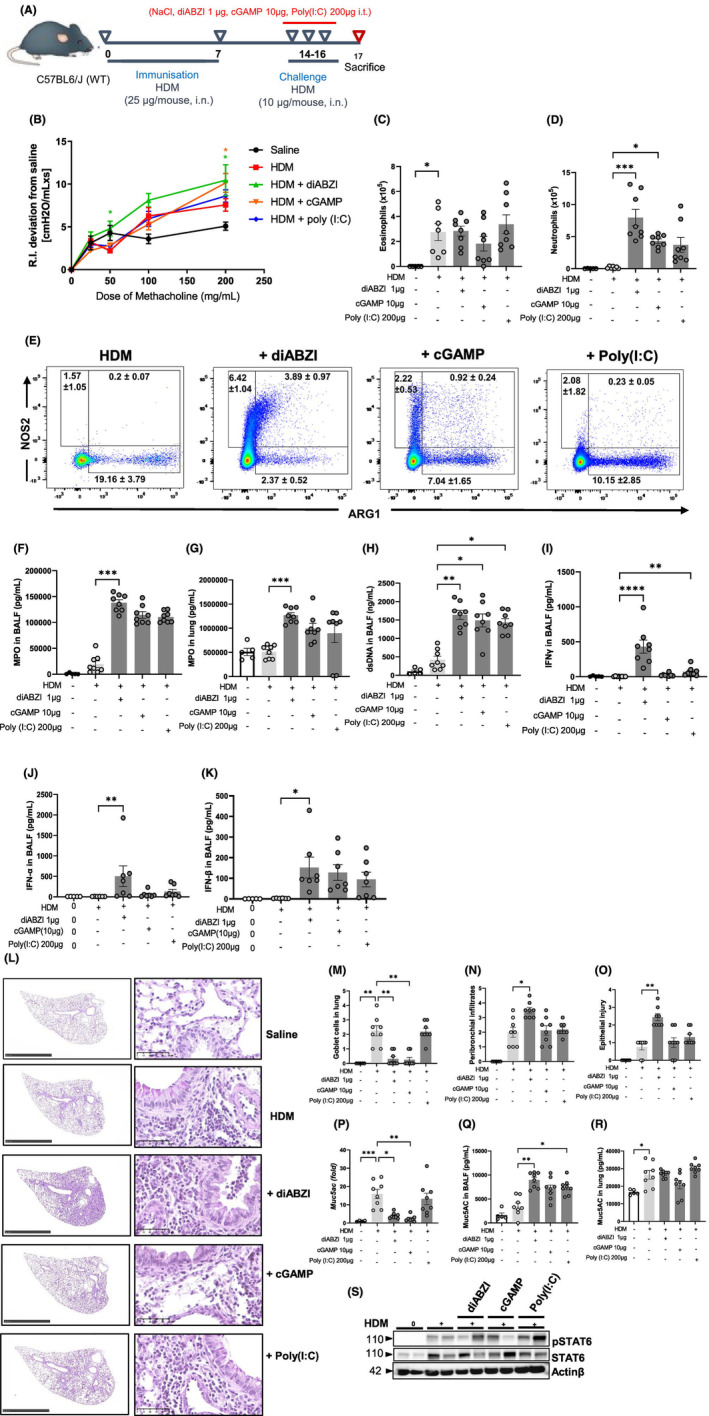
Endogenous STING agonists promote lung neutrophilia and exacerbate allergic airway inflammation. (A) Mice sensitized with HDM on day 0 and 7 (25 μg/mouse, i.n.) and challenged with HDM on day 14–16 (10 μg/mouse, i.n.) received either diABZI (1 μg/mouse, i.t.), cGAMP (10 μg/mouse, i.t.), or Poly(I:C) (200 μg/mouse, i.t.) on day 14–16, and were analyzed on day 17. (B) Airway resistance to increased doses of methacholine (50–200 mg/mL Mch) was measured 24 h after the last HDM/agonist challenges. (C) Eosinophil and (D) neutrophil counts in BAL. (E) Flow cytometry analysis of NOS2/ARG1 expressing neutrophils pre‐gated on singlet cells, and CD45^+^CD11b^+^Ly6G^+^F4/80^−^SiglecF^−^ cells. (F, G) Myeloperoxidase (MPO) concentrations in BALF and lung determined by ELISA. (H) dsDNA measured in the acellular fraction of the BAL. (I) IFNγ, (J) IFN‐α and (K) IFN‐β concentrations in BALF measured by multiplex immunoassay. (L) Histology of lung sections stained with PAS, with semi quantitative pathology scoring of (M) goblet cells, (N) peribronchial infiltrates and (O) epithelial injury. Bars, left panel: 2.5 mm, right panel: 250 μm. (P) *Muc5ac* transcripts measured by real‐time PCR (Q, R) Muc5ac protein in BALF and lung measured by ELISA. (S) Immunoblots of phospho‐STAT6 and STAT6 with Actinβ as a reference. Data were presented as mean ± SEM with *n* = 6–8 mice per group. Each point represents an individual mouse. *p < 0.05, **p < 0.01, ***p < 0.001, ****p < 0.0001 (Nonparametric Kruskal–Wallis with Dunn's post‐test).

Mice anesthetized with 2% Isoflurane (ISO‐VET) were sensitized with HDM (Stallergenes Greer) on day 0 and 7 (25 μg/mouse, i.n.) and challenged with HDM intranasally on 3 consecutive days (10 μg/mouse, i.n. on day 14–16), in the absence or together with cGAMP (at 1, 3 or 10 μg/mouse, i.t.; Invitrogen), diABZI compound 3 (at 0.1 or 1 μg/mouse, i.t.; Cayman chemicals), Poly(I:C) (at 60 or 200 μg/mouse, i.t; Invitrogen), or Budesonide (at 0.3 or 1 mg/kg, i.n.; Sigma). Mice were analyzed on day 17, 24 h after the last HDM instillation (Figure 1A).

Bronchoalveolar lavage (BAL) was performed 24 h after the last challenge by flushing lung tissue four times with 0.5 mL of cold NaCl 0.9% via tracheal intubation with a cannula. BALF was collected, cells counted and cytospins performed. The supernatant of the first lavage was collected after centrifugation and stored at −80°C for dsDNA and mediator quantifications. The left lung lobe was harvested for histology, the post caval lung for RNA extraction and qPCR analysis and the right lobes for Western blot analysis and cytokine measurement. Protein extravasation in the BALF was measured by Pierce™ BCA Protein Assay (ThermoFisher®, Massachusetts).

### 2.3 Cell culture and stimulation

Human airway epithelial cells (hAEC; Epithelix) isolated from bronchial biopsies (passage 1) were maintained in 75 cm^2^ flasks in the hAEC serum‐free culture medium (Epithelix). hAEC (passage 8) were seeded in 24‐wells plate at 5 × 10^5^ cells/well, when they reached 80% confluence. Cells were stimulated with HDM at 100 μg/mL alone or in combination with diABZI (10 μM), cGAMP (14 μM) or Poly(I:C) (100 μg/mL) diluted in hAEC serum‐free culture medium supplemented with 0.2% SVF for 2 or 24 h at 37°C and 5% CO_2_. Human airway bronchial epithelium (MUCILAIR™; Epithelix), from a single healthy or donor with asthma were maintained in MUCILAIR™ serum‐free medium (Epithelix) and stimulated on the apical face, with HDM at 100 μg/mL alone or in combination with diABZI, cGAMP or Poly(I:C) as above for 6 h. Supernatant from hAEC and MUCILAIR™ cultures were collected for mediator measurements and cells harvested for mRNA and proteins analysis.

### 2.4 Statistical analysis

Statistical analysis was performed with GraphPad Prism 9.0 software (San Diego). To determine whether the data come from Gaussian distribution, column statistic using Shapiro–Wilk normality test is performed before running the statistical analysis. Statistical significance was determined by non‐parametric Kruskal‐Wallis multiple‐comparisons tests followed by Dunn multiple comparison post‐test or two‐way ANOVA followed by Sidak post‐test as indicated in figure legends. All data are shown as mean ± SEM. Saline control data are compared with HDM group. HDM −/+ agonist groups are compared. *p* value <0.05 was considered significant. *p < 0.05, **p < 0.01, ***p < 0.001 and ****p < 0.0001.

## 3 RESULTS

### 3.1 Synthetic STING agonist diABZI triggers a neutrophilic asthma exacerbation in HDM sensitized mice

To evaluate how STING activation by agonists modulates type 2 immune response, we tested their effects during the challenge phase of HDM‐induced allergic lung inflammation in mice. We compared the effect of the natural STING agonist cGAMP, and of the potent, non‐nucleotidyl STING agonist diABZI,^16^ to the TLR3 agonist dsRNA Poly(I:C), all known to induce IRF3‐type 1 IFN response. Wild type BL6 (WT) mice sensitized with HDM on day 0 and 7 (25 μg/mouse, i.n.) and challenged on day 14–16 (10 μg/ mouse, i.n.) received either diABZI (1 μg/mouse, i.t.), cGAMP (10 μg/mouse, i.t.), or Poly(I:C) (200 μg/mouse, i.t.), doses determined from previous titrations (Figure E1–3—Figure S1), and were analyzed on day 17, 24 h after the last challenge (Figure 1A). First, we evaluated airway hyperresponsiveness (AHR), one of the hallmarks of allergic asthma known to correlate with disease severity.^17^, ^18^ There was a significant increase of AHR in HDM‐challenged WT mice at a dose of 200 mg/mL of methacholine (MCh), as compared to saline control (Figure 1B). Administration of diABZI during HDM challenge further increased AHR significantly, while cGAMP increased AHR only at 200 mg/mL of MCh, and Poly(I:C) had no effect, compared to untreated HDM‐challenged mice (Figure 1B). DiABZI and cGAMP administration had little effect on eosinophil recruitment in the airways (Figure 1C), while they strongly increased neutrophil recruitment (Figure 1D) in HDM‐challenged mice. Interestingly, diABZI and cGAMP promoted a subset of neutrophils expressing inducible nitric oxide synthase (iNOS/NOS2) corresponding to type 1 neutrophils (N1, NOS2^+^ARG1^−^) while they decreased the frequency of arginase 1 (ARG1) expressing type 2 neutrophils (N2, NOS2^−^ARG1^+^) (Figure 1E, Figure E4A–D—Figure S1). This increase in N1 neutrophils was associated with higher myeloperoxidase (MPO) in BALF and lung (Figure 1F,G), total protein extravasation (Figure E4E—Figure S1) and dsDNA release in BALF (Figure 1H). There was also an increase in macrophages, but not in lymphocytes influx in BAL (Figure E4A–C—Figure S1) or Th2 cytokines IL‐4, IL‐5 and IL‐13 (Figure E4F–K—Figure S1). The exacerbation effect was observed especially with diABZI, inducing a strong IFNγ response (Figure 1I) with CCL11, IL‐6 and TNFα release in the airways (Figure E4L–P—Figure S1). The Th2 attracting chemokines CCL17 and CCL22 were increased in the lung after HDM challenge, and CCL17 was significantly reduced after diABZI or cGAMP exposure, while CCL22 was unaffected (Figure E4 T,U—Figure S1). Further, IFNα/β, the end‐products of STING pathway activation were increased in BALF upon diABZI administration (Figure 1J,K).

STING agonists induced a narrowing of the airways with severe infiltration of inflammatory cells in the peribronchial area, epithelial injury, bronchiolitis, alveolitis and decreased goblet cells hyperplasia and production of mucus together with a decrease in *Muc5ac* transcripts in the lung compared to HDM‐challenged mice (Figure 1L–P), while TLR3 agonist caused no major changes (Figure 1L–O). Muc5AC protein concentration doubled in BALF after administration of STING or TLR3 agonists while it was essentially unaffected in the lung (Figure 1Q,R). STAT6, the transcription factor involved in *Muc5ac* gene induction and mucus hypersecretion^19^ was phosphorylated in the lung of HDM challenged mice and this was increased by STING or TLR3 agonists (Figure 1S, Figure E4Q—Figure S1).

Thus, administration of the potent STING agonist diABZI during HDM challenge augmented HDM‐induced lung resistance, together with a strong neutrophilic response characterized by neutrophil recruitment, MPO expression, protein extravasation, overexpression of Th1 cytokine IFNγ and extracellular dsDNA release in the airways.

### 3.2 NETosis and PANoptosis as main sources of airway dsDNA fueling STING‐triggered exacerbation of HDM inflammation

**FIGURE 2:**
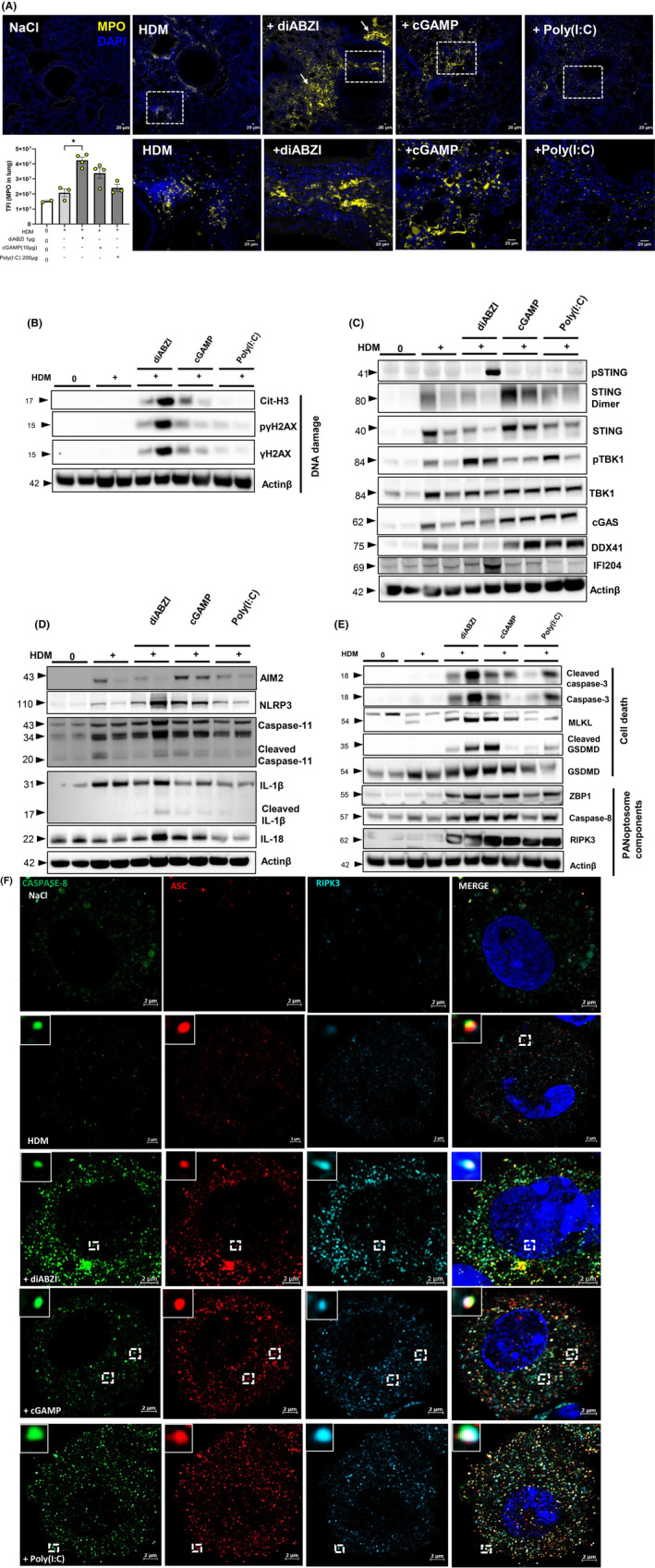
NETosis, apoptosis, pyroptosis and necroptosis (PANoptosis) as main sources of airway dsDNA exacerbating HDM‐induced lung inflammation. Mice sensitized and challenged with HDM received either diABZI, cGAMP, or Poly(I:C) as in Figure 1, and were analyzed on day 17. (A) Visualization of NETs formation in lung tissue with the staining of DNA dye DAPI (Blue) and MPO (Yellow). Bars (20 μm). Tissue fluorescence intensity (TFI) of MPO staining in lung. (B) Immunoblot highlighting DNA damage with expression of Cit‐H3, phospho‐γH2AX and γH2AX with Actinβ as a reference. (C) Immunoblot of STING axis including phospho‐STING, STING dimer, STING, phospho‐TBK1, TBK1 and DNA sensors including cGAS, DDX41 and IFI204 with Actinβ as a reference. (D) Immunoblot of inflammasome activation including AIM2, NLRP3, caspase‐11, cleaved caspase‐11, IL‐1β, cleaved IL‐1β and IL‐18 with Actinβ as a reference. (E) Immunoblot of cell death axis showing cleaved caspase‐3, caspase‐3, MLKL, cleaved GSDMD, GSDMD, ZBP1, caspase‐8, and RIPK3 with Actinβ as a reference. The analysis of cell damage, cell death and PANoptosis (B, E) was performed in the same membrane, therefore the same Actinβ control is shown. (F) Confocal microscopy showing caspase‐8 (green), ASC (red), RIPK3 (far‐red/turquoise blue) and DNA dye DAPI (cyan) in BAL cells showing the colocalization of PANoptosome components. Bars, 2 μm. *p* value ﹤0.05 was considered significant. *p ﹤ 0.05.

Extracellular dsDNA release is an active process in several chronic inflammatory diseases. Therefore, we first assessed the ability of neutrophils to form NETs.^20^, ^21^ Indeed, MPO staining demonstrated that unlike HDM‐treated mice that showed localized cellular staining, the lung of cGAMP‐ and more importantly diABZI‐challenged mice exhibited the typical morphological shape of NETs (Figure 2A), with citrullinated histone H3 (Cit‐H3) revealed by immunoblot (Figure 2B, Figure E5A—Figure S1). In contrast, Poly(I:C) did not result in the formation of NETs, with intracellular MPO staining and absence of Cit‐H3 (Figure 2A,B). DNA damage was documented by increased pγH2AX after both diABZI and cGAMP challenges, but less after Poly(I:C) administration (Figure 2B, Figure E5A—Figure S1).

NETs comprise host DNA fibers coated with cytoplasmic proteases, making host DNA available to activate DNA sensors. Indeed, cGAS, IFI204 and/or DDX41 expression increased in the lung of HDM and STING agonist challenged mice (Figure 2C, Figure E5B—Figure S1). Further, there was evidence of inflammasome activation, likely involving AIM2 and/or NLRP3, with non‐canonical caspase‐11 upregulation, resulting in cleavage of IL‐1β and IL‐18 release after STING trigger (Figure 2D, Figure E5C—Figure S1).

NETs associated molecules such as MPO, neutrophil elastase and defensins may promote lung injury, and directly induce epithelial and endothelial cell death.^20^, ^21^ HDM‐immunized and challenged mice co‐administered with STING or TLR3 agonists induced caspase 3 cleavage indicative of apoptosis in the lung tissue, but also cell death markers of necroptosis and pyroptosis with upregulation of Mixed Lineage Kinase domain Likepseudokinase (MLKL) and Gasdermin D (GSDMD), and GSDMD cleavage indicative of PANoptosis (Figure 2E, Figure E5D—Figure S1). We further assessed specific components of the macromolecular complexes regulating PANoptosis by immunoblot. Indeed, Z‐DNA binding protein 1 (ZBP1), caspase‐8 and RIPK3 were upregulated after challenge with STING or TLR3 agonists in HDM‐treated mice (Figure 2E, Figure E5D—Figure S1). We also demonstrated a co‐localization of PANoptosis components caspase‐8, ASC and RIPK3 proteins in macrophages from BAL cells (Figure 2F) which is absent in neutrophils (Figure E5E—Figure S1).

Therefore, neutrophils induced during HDM challenge with STING agonists undergo NETs formation, releasing host DNA available to activate innate immune DNA sensors, and may induce lung tissue damage and death by PANoptosis, which in turn is amplifying DNA release, thus setting the conditions for a positive feedback.

### 3.3 Glucocorticoid‐resistance of diABZI‐STING induced asthma exacerbation

**FIGURE 3:**
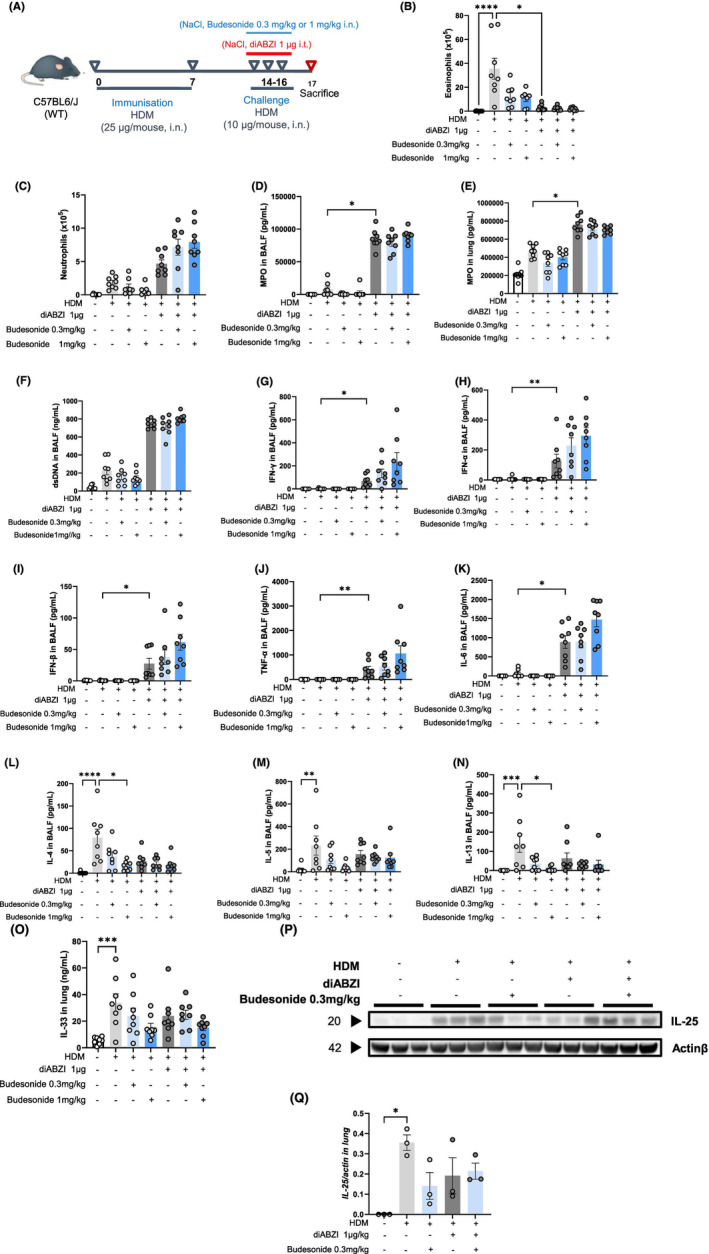
Asthma exacerbation induced by diABZI‐STING glucocorticoid resistance. (A) Wild‐type mice (WT) were sensitized with HDM on day 0 and 7 (25 μg/mouse, i.n.), challenged with HDM on day 14–16 (10 μg/mouse, i.n.) without or with diABZI (1 μg/mouse, i.t.) and Budesonide (0.3 or 1 mg/kg), and analyzed on day 17. (B) Eosinophil and (C) neutrophil counts in BAL. (D, E) Myeloperoxidase (MPO) concentrations in BALF and lung determined by ELISA. (F) dsDNA measured in the acellular fraction of the BALF. (G) IFNγ, (H) IFN‐α and (I) IFN‐β concentrations in BALF measured by multiplex immunoassay. (J, K) TNF‐α and IL‐6 in BALF determined by ELISA. (L) IL‐4, (M) IL‐5 and (N) IL‐13 concentrations in BALF measured by ELISA. Epithelial‐derived alarmins (O), IL‐33, (P, Q) Immunoblot of IL‐25 protein and quantification. Data were presented as mean ± SEM with *n* = 8 mice per group. Each point represents an individual mouse. *p < 0.05, **p < 0.01, ***p < 0.001, ****p < 0.0001 (Nonparametric Kruskal–Wallis with Dunn's post‐test).

Severe asthma fail to respond to conventional anti‐asthma therapy such as corticosteroids. To assess whether the model of asthma exacerbation induced by the STING agonist diABZI is glucocorticoid‐resistant, diABZI+HDM‐treated mice were treated with Budesonide (0.3 or 1 mg/kg i.n.) during the challenge phase (Figure 3A). Budesonide treatment had no effect on diABZI‐induced asthma exacerbation, which is characterized by decreased eosinophils (Figure 3B) and conversely increased neutrophils (Figure 3C) associated with higher myeloperoxidase (MPO) (Figure 3D,E) in BALF and lung, dsDNA release in BALF (Figure 3F), CXCL1 in BALF and lung (Figure E6 A,D—Figure S1), CXCL10 and CCL11 in BALF (Figure E6 B,C—Figure S1). Budesonide treatment tended to increase IFNγ (Figure 3G), type I IFNα/β (Figure 3H,I) and pro‐inflammatory cytokines TNFα and IL‐6 in BALF and lung (Figure 3J,K and Figure E6 E,F—Figure S1). We further investigated the effect of Budesonide on type 2 cytokine/chemokine production. While Budesonide treatment decreased the levels of IL‐4, IL‐5 and IL‐13 in BALF, IL‐33, IL‐25 as well as CCL17 and CCL22 in the lungs of HDM‐treated mice (Figure 3L–Q and Figure E6 G–J—Figure S1), it had no significant effect on these cytokines during the exacerbation state induced by concomitant administration of diABZI and HDM (Figure 3L–Q).

Thus, the features of diABZI‐induced exacerbated responses to HDM were unresponsive to Budenoside.

### 3.4 STING dependence of the neutrophilic exacerbation of HDM‐induced response

**FIGURE 4:**
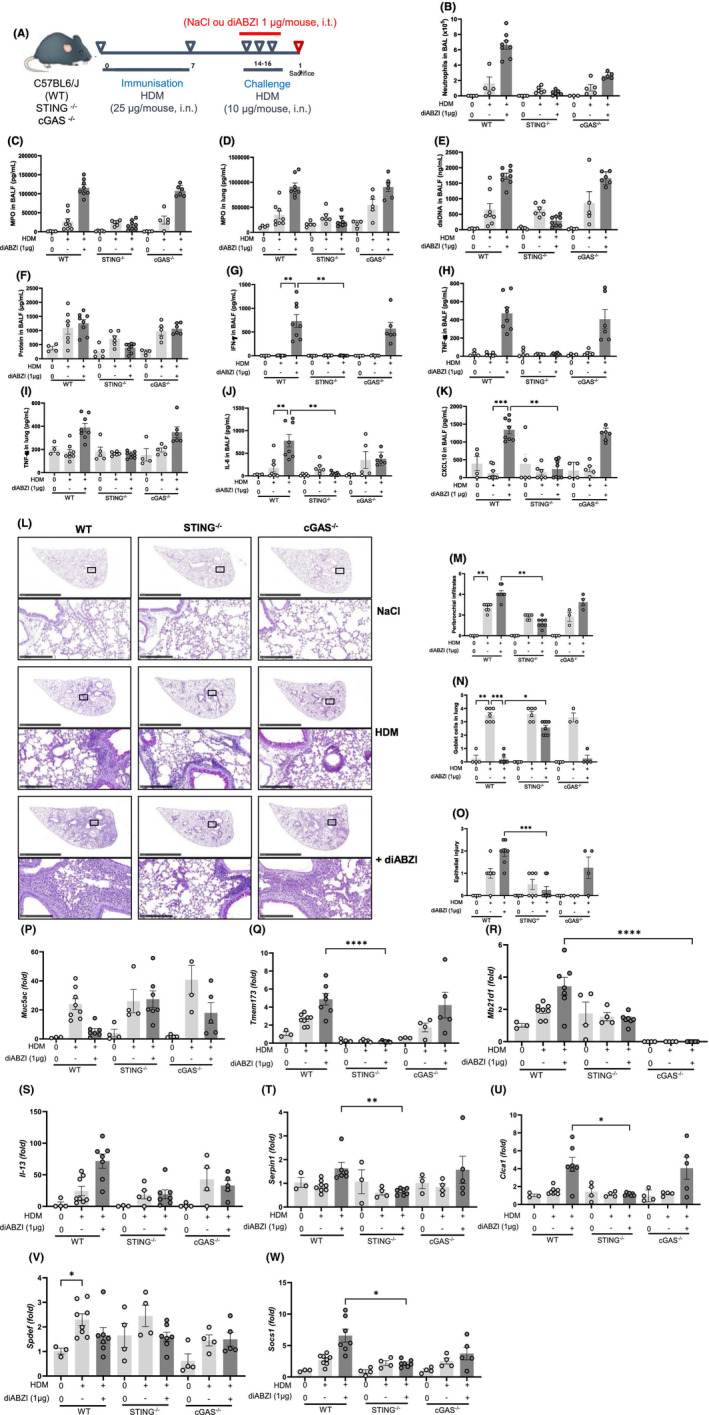
STING dependence of the neutrophilic airway inflammation exacerbation. (A) Wild‐type (WT), STING^−/−^ or cGAS^−/−^ mice were sensitized with HDM on day 0 and 7 (25 μg/mouse, i.n.), challenged with HDM on day 14–16 (10 μg/mouse, i.n.) without or with diABZI (1 μg/mouse, i.t.), and analyzed on day 17. (B) Neutrophils count in BAL. (C, D) MPO concentration in BALF and lung determined by ELISA. (E) dsDNA concentration in the BAL acellular fraction. (F) Protein concentration in BALF. (G) IFN‐γ in BALF determined by multiplex immunoassay. (H, I) TNF‐α in BALF and lung determined by ELISA. (J) IL‐6 and (K) CXCL10/IP‐10 concentration in BALF measured by ELISA. (L) Histology of lung tissues stained with PAS of WT, STING^−/−^ and cGAS^−/−^ mice, with semi quantitative pathology scoring of (M) peribronchial infiltrates, (N) goblet cells and (O) epithelial injury. Bars, upper panel: 2.5 mm, lower panel: 250 μm. (P–W) *Muc5ac, Tmem173, Mb21d1, Il‐13 Serpin1, Clc1, Spdef* and *Socs1* transcripts measured by real‐time qPCR. Data were presented as mean ± SEM with *n* = 6 ~ 8 mice per group. Each point represents an individual mouse. *p < 0.05, **p < 0.01, ***p < 0.001, ****p < 0.0001 (Nonparametric Kruskal–Wallis with Dunn's post‐test).

To evaluate the STING specificity of diABZI induced asthma exacerbation, response to the extracellularly released dsDNA, mice deficient for STING (STING^−/−^) or cGAS (cGAS^−/−^) were sensitized and challenged with HDM with or without diABZI as above (Figure 4A). DiABZI‐induced increase of neutrophils was absent in the airways of HDM‐challenged STING^−/−^ mice, and reduced in cGAS^−/−^ mice (Figure 4B). Conversely, diABZI reduced HDM‐induced eosinophils, and this was less prominent in STING^−/−^ mice (Figure E7B—Figure S1). HDM‐induced rise in macrophages increased after DiABZI in wild‐type mice and less so in STING^−/−^ mice (Figure E7C—Figure S1). In addition, diABZI‐induced increase of MPO and extracellular dsDNA (Figure 4C–E), or protein extravasation, IFNγ, TNF, IL‐6, and CXCL10 (Figure 4F–K) release were abolished in the airways of HDM‐challenged STING^−/−^ mice, while they were barely affected in cGAS^−/−^ mice. Because allergic airway inflammation triggers Th2 immune response, we evaluated Th2 cytokines and found increased IL‐4 in the BALF of HDM‐challenged mice (Figure E7E—Figure S1), with a trends toward increased IL‐5, and *Il4* and *Il5* transcripts in the lung that did not reach statistical significance (Figure E7F,H,I—Figure S1). DiABZI further increased Th2 cytokine IL‐13 and chemokines CCL11 and CCL24 in the airways, and this was absent in STING^−/−^ and reduced in cGAS^−/−^ mice (Figure E7 G,J–L—Figure S1). Pulmonary CXCR2^+^ and CXCR4^+^ expressing neutrophils subsets increased after diABZI challenge were reduced in STING^−/−^ and cGAS^−/−^ mice (Figure E7 O–R—Figure S1).

The increased leukocyte infiltration, epithelial injury, with decreased goblet cells, mucus staining (Figure 4L–O) and *Muc5ac* transcript expression (Figure 4P) after diABZI administration in HDM‐challenged WT mice were abolished in STING^−/−^ mice, while they were barely affected in cGAS^−/−^ mice. DiABZI and HDM co‐administration upregulated *Tmem173* (STING) transcript expression in WT and cGAS^−/−^ mice (Figure 4Q), while the increase in *Mb21d1* (cGAS) transcripts was abolished in STING^−/−^ mice (Figure 4R).

IL‐13 mRNA expression, a Th2 cytokine necessary for mucus metaplasia, increased after diABZI and HDM co‐administration in WT mice, while it was barely affected in STING^−/−^ or cGAS^−/−^ mice. IL‐13 binding triggers a STAT6‐dependent transcriptional program involving the activation of Serpin1, Spdef and Clca1 (protein calcium‐activated chloride channel 1) whose transcripts were overexpressed after diABZI administration in HDM‐challenged WT mice but not in STING^−/−^ mice (Figure 4S–V). On the other hand, the transcripts for *Socs1*, a negative regulator of *Muc5ac* expression,^22^ was overexpressed after diABZI co‐administration in WT mice but not in STING^−/−^ mice (Figure 4W), in line with the fact that STING activation was shown to enhance SOCS1 expression.^23^

Thus, diABZI‐induced asthma exacerbation is mediated by STING pathway activation in terms of neutrophil recruitment, release of extracellular dsDNA and pro‐inflammatory cytokines in the airways, increased epithelial injury with decreased goblet cells and mucus production, while the involvement of cGAS is more limited.

### 3.5 STING‐deficient macrophages and granulocytes mitigate asthma exacerbation in vivo

**FIGURE 5:**
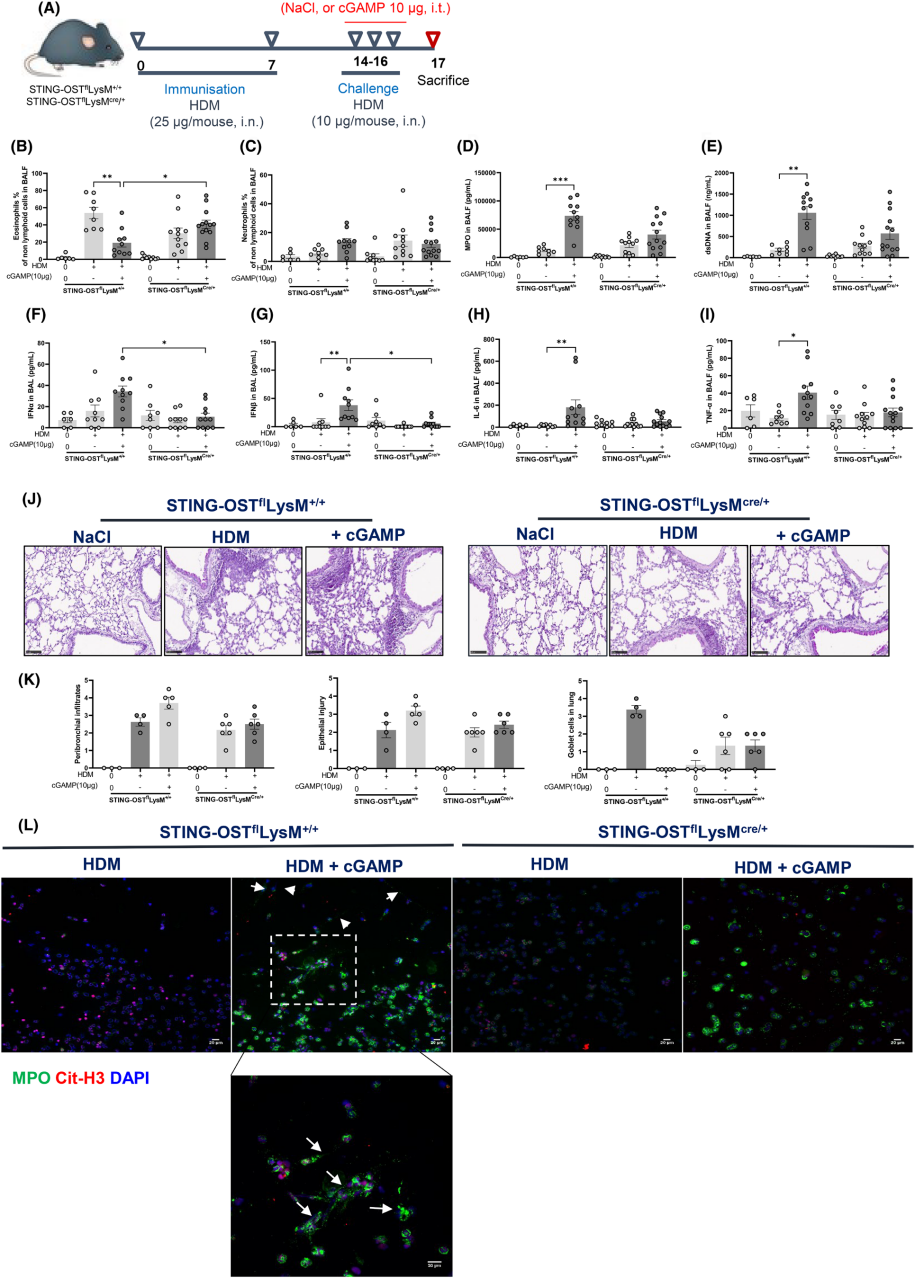
STING‐deficient macrophages and granulocytes mitigates asthma exacerbation in vivo in the lung. (A) STING‐OST^fl^LysM^cre/+^ or STING‐OST^fl^LysM^+/+^ mice were sensitized with HDM on day 0 and 7 (25 μg/mouse, i.n.), challenged with HDM on day 14–16 (10 μg/mouse, i.n.) without or with cGAMP (10 μg/mouse, i.t.), and analyzed on day 17. (B) Eosinophils and (C) Neutrophils counts in BAL. (D) MPO concentration in BALF measured by ELISA. (E) dsDNA measured in the acellular fraction of the BAL. (F) Concentration of IFN‐α and (G) IFN‐β in BALF measured by luminex immunoassay. (H) IL‐6 concentration (I) and TNF‐α concentration in BALF measured by ELISA. (J) Lung tissue histology with PAS staining of STING‐OST^fl^LysM^cre+^ or STING‐OST^fl^LysM^+/+^ mice with semi quantitative pathology scoring of (K) peribronchial infiltrates, epithelial injury and goblet cells. (L) Visualization of NETs in BAL with the staining of DNA dye DAPI (blue), MPO (Green), CitH3 (Red). Bars, 20 μm. Data were presented as mean ± SEM with *n* = 8 ~ 13 mice per group. Each point represents an individual mouse. *p < 0.05, **p < 0.01, ***p < 0.001 (Nonparametric Kruskal–Wallis with Dunn's post‐test).

Neutrophilic inflammation is observed during asthma exacerbation but also in the airways of patients with severe asthma.^24^ In addition to neutrophils, macrophages are one of the main lung cell types responsible for the uptake of cGAMP in vivo.^25^ We investigated the contribution of both cell types in mice by generating conditional STING deficient mice. We crossed mice carrying STING‐OST floxed alleles (STING‐OST^fl^) with LysM^cre^ transgenic mice expressing Cre under the control of the LysM promotor to obtain STING‐OST^fl^LysM^cre/+^ mice deficient for STING gene in myeloid cells such as granulocytes and macrophages, as well as STING‐OST^fl^LysM^+/+^ control mice (Figure E8 A–D—Figure S1). Administration of cGAMP during HDM challenges resulted in a reduction of eosinophils and an increase in total cells, neutrophils, MPO and self dsDNA in BALF of STING‐OST^fl^LysM^+/+^ control mice (Figure 5 A–E and Figure E8 E,G—Figure S1). These parameters were partially reduced in STING‐OST^fl^LysM^cre/+^ suggesting that myeloid cells, but also other cell types contribute to shape the neutrophilic response after STING trigger (Figure 5A–E). cGAMP induced an increase in type I IFNα/β, as well as pro‐inflammatory cytokines TNFα and IL‐6 production in HDM‐treated STING‐OST^fl^LysM^+/+^ control mice that were partially reduced in STING‐OST^fl^LysM^cre/+^ (Figure 5F–I). The increase in peribronchial leukocytes infiltration and epithelial injury following cGAMP administration in STING‐OST^fl^LysM^+/+^ mice was abrogated in STING‐OST^fl^LysM^cre/+^ (Figure 5J,K). Moreover, protein extravasation in BALF was reduced in STING‐OST^fl^LysM^cre/+^ mice in comparison with control mice (Figure E8F—Figure S1), which was suggestive of a less severe barrier disruption. Neutrophils infiltrating the lung of mice lacking STING in granulocytes/macrophages, were tested for their ability to form NETs. BAL cells from STING‐OST^fl^LysM^+/+^ control mice challenged with HDM and cGAMP showed the formation of a weblike structure positive for MPO and Cit‐H3 staining (Figure 5L), suggestive of NET formation. In contrast, this process was reduced in STING‐OST^fl^LysM^cre/+^ group (Figure 5L), in line with the reduced histopathological damage observed.

Thus, these data indicate that granulocytes/macrophages, but also other cell types, contribute to the development of STING‐dependent neutrophilic asthma exacerbation in vivo.

### 3.6 Epithelial cells respond to diABZI and may contribute to STING agonist‐induced neutrophilic asthma exacerbation

**FIGURE 6:**
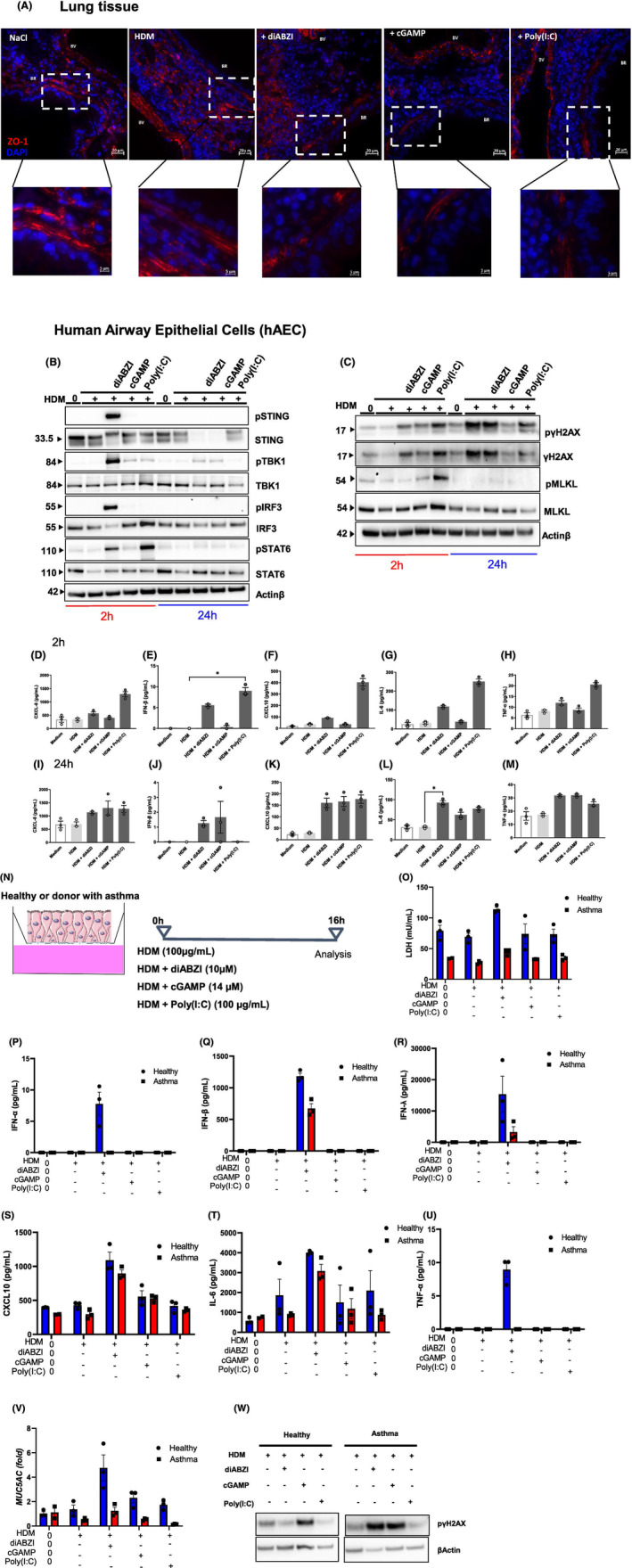
Epithelial cells contribute to the process of STING agonists‐induced neutrophilic asthma exacerbation. (A) Immunofluorescence staining of Zonulin‐1 (ZO‐1) (RED) in lung tissue sections from mice treated by HDM and challenged or not with diABZI, cGAMP, or Poly(I:C) as in Figure 1, counterstained with DAPI (Blue). Upper panel: 20 μm, lower panel: 5 μm. Immunoblots of human airway epithelial cell (hAEC) stimulated with HDM at 100 μg/mL alone or in combination with diABZI (10 μM), cGAMP (14 μM) or Poly(I:C) (100 μg/mL) for 2 or 24 h showing (B) STING pathway activation including phospho‐STING, STING, phospho‐TBK1, TBK1, phospho‐IRF3, IRF3, phospho‐STAT6 and STAT6, with Actinβ as a reference. (C) DNA damage and cell death axis including phospho‐γH2AX, γH2AX, phospho‐MLKL, MLKL and Actinβ as a reference. (D‐M) Cytokine concentrations of (D, I) IL‐8/CXCL8, (E, J) IFN‐β, (F, K) IP‐10/CXCL10, (G, L) IL‐6 and (H, M) TNF‐α in cell culture supernatant were measured by multiplex immunoassay at 2 h (D‐H) and 24 h (I–M). Data were presented as mean ± SEM with *n* = 3 independent wells from the same donor. (N) Human airway epithelium (MUCILAIR™) from a single healthy donor or patient with asthma were restimulated on the apical face, with HDM at 100 μg/mL alone or in combination with diABZI at 10 μM, cGAMP at 14 μM or Poly(I:C) at 100 μg/mL. At 6 h post‐stimulation, (O) Lactate dehydrogenase (LDH) concentration was determined in the supernatant. (P–U) Multiplex immunoassay of IFN‐α (P), IFN‐β (Q), IFN‐λ (R), IP‐10/CXCL‐10 (S), IL‐6 (T) and TNF‐α (U) concentrations in the supernatant. (V) *MUC5AC* transcripts measured by real‐time PCR. (W) Immunoblot from epithelial cell homogenates from healthy and patients with asthma showing the expression of phospho‐γH2AX with Actinβ as a reference. Data were presented as mean ± SEM with *n* = 3 independent wells from the same donor. *p < 0.05. (D–M) Nonparametric Kruskal–Wallis with Dunn's post‐test. (O–V) Two‐way ANOVA followed by Sidak multiple comparison test.

Allergens cause airway epithelium damage and structural changes including downregulation of group I tight junction and extracellular matrix (ECM) expression which are often associated with asthma exacerbations and severity.^26^, ^27^, ^28^ The transmembrane proteins Zona‐occludens‐1 (ZO‐1) are scaffold proteins important for cell–cell adhesion in healthy tissue and main regulators of epithelial permeability in allergic lung inflammation.^27^ After confirming the role of diABZI on immune cells infiltration, we investigated its effect on human airway epithelium. HDM led to ZO‐1 downregulation in lung tissue compared to saline control (Figure 6A and Figure E9 B—Figure S1), as expected.^27^ STING agonists DiABZI and cGAMP, and Poly(I:C) further downregulated ZO‐1 expression as evidenced by immunofluorescence staining (Figure 6A). To evaluate the ability of diABZI to activate STING pathway in epithelial cells, human airway epithelial cells (hAEC) from bronchial biopsies were stimulated for 2 h or 24 h with HDM alone or in combination with diABZI, cGAMP or Poly(I:C). There was evidence of STING pathway activation with the detection of phosphorylated STING, TBK1 and IRF3 when hAEC were co‐stimulated with diABZI detectable at an early stage, 2 h post stimulation (Figure 6B, Figure E9A—Figure S1). cGAMP and Poly(I:C) co‐stimulation also induced TBK1 phosphorylation at 2 h, that persisted after 24 h with diABZI and cGAMP. Moreover, there was evidence of epithelial DNA damage in hAEC, revealed by phosphorylation of γH2AX at 2 h and 24 h post stimulation (Figure 6C, Figure E9C—Figure S1), and some activation of necroptosis with the phosphorylation of MLKL at 2 h (Figure 6C, Figure E9A—Figure S1). Activating STING with diABZI in cells sensitized with HDM yielded secretion of inflammatory cytokines 2 h post stimulation that was sustained after 24 h, such as CXCL8, IFNβ, CXCL10, IL‐6 and TNFα (Figure 6D–M).

To further characterize the contribution of epithelial barrier, air liquid interface (ALI) culture of bronchial epithelial cells isolated from the lung of healthy donor and patient with asthma were performed and stimulated for 16 h with HDM alone or in combination with diABZI, cGAMP or Poly(I:C) (Figure 6N). DiABZI induced an increased release of lactate dehydrogenase (LDH), IFNα, IFNβ, IFNλ, CXCL10, IL‐6 and TNFα protein and overexpression of *MUC5AC* transcript in healthy epithelial cells sensitized with HDM (Figure 6O–V), and less in epithelial cells from patient with asthma (Figure 6O–V). Further, diABZI induced epithelial DNA damage evidenced by the phosphorylation of γH2AX in epithelial cells from patient with asthma, compared to healthy cells (Figure 6W).

Thus, diABZI affected airway epithelium cells by activating STING pathway, impairing tight junctions, and inducing epithelial DNA damage, necroptosis and secretion of inflammatory cytokines, that may further fuel the lung inflammation.

### 3.7 Viral infection of human bronchial epithelial cells from patients with asthma aggravates inflammation and weakens epithelial barrier both in vitro and in vivo

To ascertain the clinical relevance of these findings, we first analyzed the different expression of STING and PANoptosis pathways in PBMC from healthy controls or patients with severe asthma (Figure E10—Figure S1). Indeed, host double‐stranded DNA released by NETosis promote rhinovirus‐induced asthma exacerbations.^29^ Therefore, we analyzed DNA‐sensing, PANoptosis, tight junction, and neutrophils gene signatures from two independent studies addressing rhinovirus (RV) infection in patients with asthma, either in vitro in differentiated primary bronchial epithelial cells (HBECs) from healthy and individuals with asthma,^30^ or in vivo in bronchial brushing from intranasally RV infected healthy controls and patients with asthma^31,32^(Supl Figure E11).

**FIGURE 7:**
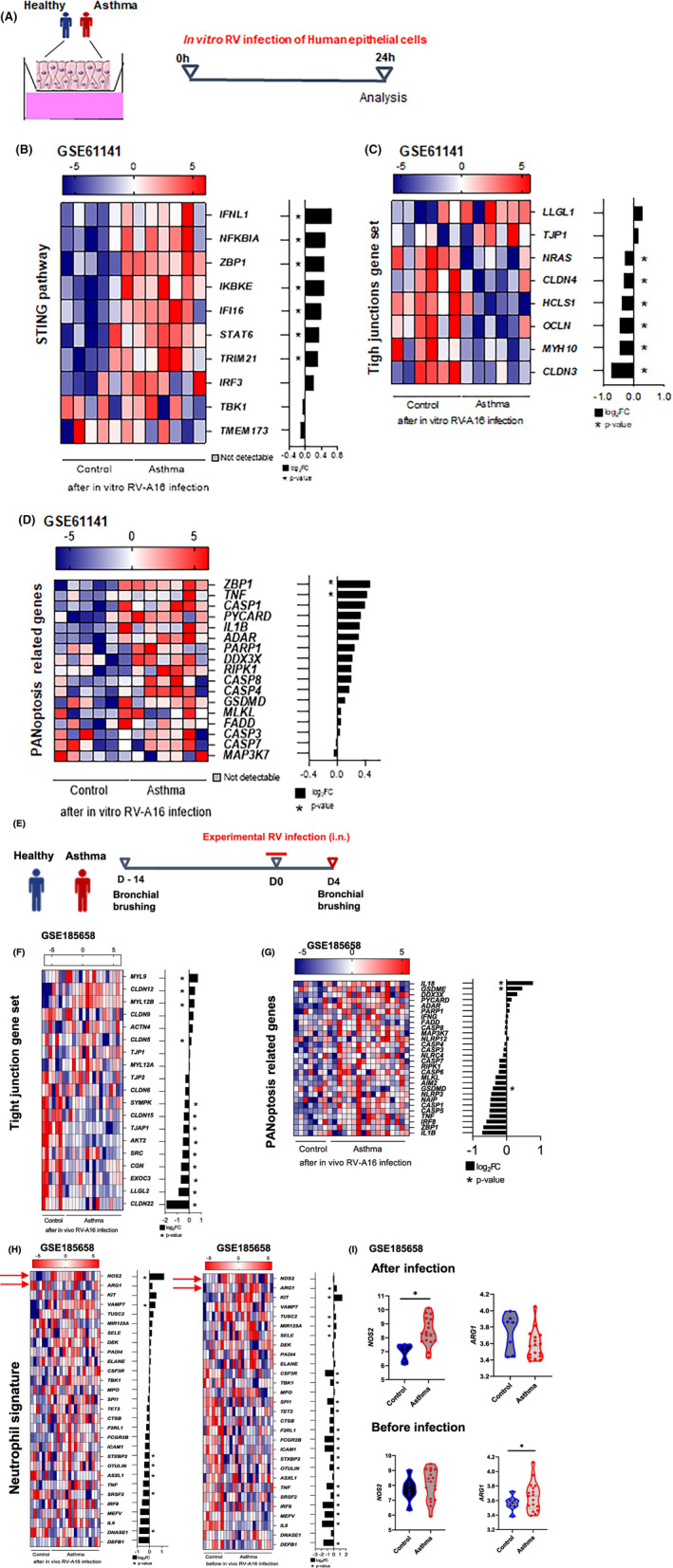
Upregulation of DNA sensing and PANoptosis pathways upon rhinovirus infection in patients with asthma. (A) Human bronchial epithelial cells (HBECs) from patients with asthma (*n* = 6) and healthy controls (*n* = 6), were infected with RV‐A16 for 24 h, and subjected to transcriptome analysis. (B‐E) Heatmaps presented together with the corresponding log2 fold change (FC) expression changes (black bars) of (B) STING pathway‐related genes, (C) Tight junction genes set, (D) PANoptosis‐related genes, after in vitro RV‐A16 infection in controls compared to HBEC from patients with asthma. Transcriptomic data were processed with the workflow available on https://github.com/uzh/ezRun. (E) Experimental in vivo RV‐A16 infection in patients with asthma (*n* = 17) and healthy individuals (*n* = 7): Transcriptomic analysis of bronchial brushings 14 days before and 4 days after in vivo infection. (F–G) Heatmaps presented together with the corresponding log2 fold change (FC) expression changes (black bars) of (F) Tight junction genes set, (G) PANoptosis‐related genes, after in vivo RV‐A16 infection in controls compared to patients with asthma. (H) Heatmaps of neutrophilic signature before (right panel) and after (left panel) infection in controls compared to patients with asthma. (I) Violin plots representing gene expressing of *NOS2* and *ARG1* in healthy individual and patients with asthma, before (lower set) and after (Upper set) infection. Data was analyzed with Bioconductor microarray analysis workflow [https://www.bioconductor.org/packages/release/workflows/vignettes/arrays/inst/doc/arrays.html]. All Heatmaps display normalized gene expression across the groups (row normalization). Asterisks demonstrate significantly changed genes with threshold *p* < 0.05. *p*‐value: * < 0.05. Publicly available data under accession number: GSE185658 and GSE61141. Source data are provided as Source Data files.

Human epithelial cells from patients with asthma stimulated in vitro with RV‐A16 (Figure 7A) showed an upregulation of genes involved in DNA‐sensing and STING pathway (Figure 7B) such as the DNA sensors ZBP1, IFI16 and the cytosolic TRIM21. *IRF3* responsible of type I/III IFNs induction and anti‐viral defense was also upregulated in the cells of these patients, concomitant with *IFN‐λ* expression, while STING itself was not significantly downregulated. Analysis of tight‐junction gene sets (Figure 7C) revealed the reduced expression of a whole cluster of genes such as *OCLN, CLDN3, CLDN4, NRAS, HCLS1, MYH10* in virus‐infected cells from patients with asthma in comparison to the controls. Moreover, analysis of PANoptosis related genes set revealed the upregulation and a tendency to upregulation of several components of the PANoptosome such as the pivotal *ZBP1*, *PYCARD*, *CASPASE‐8*, *RIPK1*, *CASPASE‐1*, and *FADD* after viral infection of the cells from patients with asthma compared to the controls (Figure 7D), which was not visible before RV infection (Figure E11 A–D—Figure S1). Mucus genes such as *MUC5AC* and *MUC5B* were also upregulated (Figure E11 E,F—Figure S1).

Bronchial brushings from patients with asthma experimentally infected in vivo with RV‐A16^31^ (Figure 7E) showed a decrease in a group of tight junction‐related gene such as *CLDN15, TJAP1, AKT2, SRC, CGN, EXOC3, LLG2, CLDN22*, compared to similarly infected healthy controls, while *CLDN12* and *MYL12B* genes were increased (Figure 7F). PANoptosis components showed a tendency toward an increase 4 days after in vivo infection in patients with asthma compared to controls (Figure 7G), in contrast to the non‐infected controls and patients with asthma, where the PANoptosis components were downregulated (Figure E11 G–I—Figure S1). Moreover, the gene expression of IL‐18 (*IL18*) was higher after infection in patients with asthma compared to healthy individuals (Figure 7G), in line with inflammasome activation.^32^ Interestingly, the NOS2/ARG1 gene expression balance was disrupted in bronchial brushings. *NOS2* was significantly increased after infection in patients with asthma compared to the controls, while *ARG1* was downregulated in patients with asthma (Figure 7H,I). Overall, these analyses show an involvement of DNA sensing pathway, weakening of the epithelial barrier and upregulation of pro‐inflammatory markers such as *NOS2* after RV infection in patients with asthma.

## 4 DISCUSSION

Asthma exacerbations are complex events, triggered by various factors, including respiratory infections, allergens, and environmental pollutants.^33^, ^34^ The cGAS/STING pathway plays a critical role in the innate immune response to microbial DNA, leading to the production of type I IFNs and other inflammatory cytokines.^35^ Respiratory viruses such as rhinovirus and influenza often cause neutrophil‐dominant inflammation, characterized by elevated neutrophils in the airways and increased production of neutrophil‐attracting chemokines in asthmatic patients.^36^, ^37^ This form of inflammation is frequently observed in treatment‐refractory asthma exacerbations, where standard therapies fail to achieve adequate control.^38^

Here we show that STING activation during type 2 allergic response challenges triggers asthma exacerbation, replicating key characteristics of severe asthma and epithelial damage. Administering the synthetic STING agonist diABZI during an HDM challenge in mice exacerbates asthma by heightening epithelial damage, promoting dsDNA release, causing DNA damage, triggering cell death through NETosis and PANoptosis, and increasing neutrophil influx in the lungs. This neutrophilic asthma exacerbation is unaffected by budesonide, thus validating the corticosteroids resistance of the STING‐dependent exacerbation.

The occurrence of PANoptosis is a downstream consequence of STING activation, and we demonstrate a link between NETs and PANoptosis markers in the lung. However, it would be interesting in future studies to gain insight on the precise molecular mechanisms involved in these processes.

Co‐stimulation of human epithelial cells with diABZI and HDM induces IL‐6, TNFα, type I IFNα/β, and type III IFNλ release in vitro, causing DNA damage and cell death. These findings suggest that STING agonists exacerbate asthma by promoting dsDNA release after cell death, triggering a Th1 response and neutrophilic asthma exacerbation.

Our findings are consistent with several established mechanisms of asthma exacerbation, confirming their importance in a relevant human study. Host DNA released during NETosis activates the STING pathway, promoting Type I IFN production. Type I IFNs are crucial in linking viral infections including rhinovirus infection to asthma exacerbations. The inflammatory response they mediate can lead to increased airway hyperresponsiveness and asthma symptoms.^1^

STING agonists lead to a reduction of goblet cell numbers and of mucus transcript in the lung of HDM‐challenged mice, together with a strong release of mucus protein in the airways. A possible explanation is that diABZI induced epithelial barrier damage, documented by epithelium cell tight junction ZO‐1 protein expression, leading to Goblet cells shedding and release of their mucus content, which may clog the airspace and contribute to the exacerbation of HDM‐induced lung resistance.

However, not all treatment‐refractory exacerbations are neutrophil‐dominant. Indeed, a subset of patients with severe asthma exhibits a type 2 helper T cell inflammatory profile (Th2‐high), characterized by elevated eosinophils, IgE, and cytokines such as IL‐4, IL‐5, and IL‐13.^39^, ^40^ Th2‐high asthma is often associated with allergic triggers and a distinct molecular and clinical phenotype compared to neutrophil‐dominant asthma. Respiratory infections can also play a significant role in exacerbation in the context of Th2‐high severe asthma. Indeed, viral infections may exacerbate Th2‐high inflammation by inducing epithelial cell‐derived cytokines such as IL‐25, IL‐33, and thymic stromal lymphopoietin (TSLP), which further activate and recruit type 2 helper T cells and eosinophils.^31^, ^41^

Neutrophils display remarkable plasticity, their functional phenotype being influenced by the local microenvironment. In the inflamed asthmatic airway, factors such as cytokine milieu, microbial products, and tissue‐derived signals can modulate neutrophil behavior. For instance, exposure to cytokines such as GM‐CSF, IL‐17, and IFNγ may skew neutrophils towards a pro‐inflammatory state, while signals such as IL‐10 and TGFβ promote anti‐inflammatory properties.^42^

Here, we demonstrate that STING agonists strongly induced N1 (NOS2^+^ARG1^−^) subset. This phenotypic shift from HDM baseline N2 (ARG1^+^NOS2^−^) toward N1 pro‐inflammatory after STING agonist challenge, may modify neutrophil function and trigger NETs formation process, in part responsible of lung tissue damage. Conversely, the N2 (ARG1^+^ NOS2^−^) profile of Poly(I:C)‐induced neutrophils may explain the lack of NETs in the lung of Poly(I:C) treated HDM challenged mice. NOS2 and ARG1 are important enzymes regulating both macrophage and neutrophil functions and markers of alternative activation.^43^, ^44^ NOS2 has multiple roles in the airways, its expression being highest in severe asthma, while ARG1 decreases.^45^, ^46^, ^47^ We report increased NOS2 expression in RV‐A16 infected patients with asthma, as compared to healthy controls. Further, transcriptomic analyses of RV‐A16 infected cells from patients with asthma also indicated an involvement of DNA sensing, PANoptosis, and weakening of the epithelial barrier. Our study is the first to demonstrate the induction of this particular subset of N1, NOS2^+^ARG1^−^neutrophils following STING activation.

Neutrophils exhibit dual roles in neutrophilic severe asthma, with pro‐inflammatory and anti‐inflammatory subsets contributing to disease exacerbation and resolution, respectively. Despite their major contribution in our models, neutrophils express critically low levels of STING and cGAS.^48^ This may mean that they are not the primary cells responding to STING agonists. Instead, it is highly possible that alveolar macrophages known for their high levels of STING expression or epithelial cells are the actual first cells responding and initiating STING priming, followed by neutrophils as responder cells.

In conclusion, the present findings indicate that uncontrolled STING activation, e.g. after rhinovirus infection, may drive neutrophilic exacerbation in patients with asthma, and shift the immune response from a classical Th2‐high response to a mixed Th1/Th2 response. Therefore, considering STING inhibitors as therapeutic strategy, alone or in combination with other treatments, may improve the condition of patients with severe asthma.

## AUTHOR CONTRIBUTIONS

Y.M.‐N. performed most experiments with the assistance of E.C., I.M., R.B., C.V., F.S. and S.R. B.M. generated the STING‐OST^fl^ mouse model. Y.M.‐N., D.T., B.R. and V.F.Q. conceived the project and designed the experiments, Y.M.‐N., D.T., B.R., V.F.Q., G.V.L.S., M.R.E., D.J.J., S.L.J., M.S. and U.R. analyzed and interpreted the data. Y.M.‐N, D.T., B.R. and V.F.Q. wrote the manuscript. All authors had the opportunity to discuss the results and comment on the manuscript.

## FUNDING INFORMATION

This work was supported by CNRS, University of Orleans, ‘Fondation pour la Recherche Médicale’ (FRM EQU202003010405), Region Centre‐Val de Loire (ExAsPIR17 N° 2021‐00144721), European Regional Development Fund in Region Centre‐Val de Loire (FEDER EUROFeri N° EX010381, TARGET‐Ex N° EX016008, and Exposome&Inflammation N° 2024‐00012066), ANR‐10‐INSB‐07 and CIPHE supported by the “investissement d'avenir programme PHENOMIN” (French National infrastructure for mouse Phenogenomics). The human in vivo work was supported by the European Research Council (ERC FP7 Advanced grant number 233015); a Chair from Asthma UK (CH11SJ); the Medical Research Council Centre (grant number G1000758); National Institute of Health Research (NIHR) Biomedical Research Centre (grant number P26095); Predicta FP7 Collaborative Project (grant number 260895); and the NIHR Biomedical Research Centre at Imperial College London.

## CONFLICT OF INTEREST STATEMENT

M.S. reports research grants from Swiss National Science Foundation (nr 310030_189334/1), Novartis Foundation for Medical‐Biological Research, GSK, and Stiftung vorm. Buendner Heilstaette Arosa; speaker's fee from AstraZeneca; voluntary positions in the European Academy of Allergy and Clinical Immunology (EAACI) as Executive Board member and Basic and Clinical Immunology Section Chair. S.L.J. reports grants/contracts from European Research Council ERC FP7 grant number 233015, Chair from Asthma UK CH11SJ, Medical Research Council Centre grant number G1000758, NIHR Biomedical Research Centre grant number P26095, Predicta FP7 Collaborative Project grant number 260895, NIHR Emeritus NIHR Senior Investigator; consulting fees from Lallemand Pharma, Bioforce, resTORbio, Gerson Lehrman Group, Boehringer Ingelheim, Novartis, Bayer, Myelo Therapeutics GmbH; patents issued/licensed: Wark PA, Johnston SL, Holgate ST, Davies DE. Anti‐virus therapy for respiratory diseases. UK patent application No. GB 0405634.7, 12March 2004. Wark PA, Johnston SL, Holgate ST, Davies DE. Interferon‐Beta for Anti‐Virus Therapy for Respiratory Diseases. International Patent Application No. PCT/GB05/50031, 12 March 2004. Davies DE, Wark PA, Holgate ST, Johnston SL. Interferon Lambda therapy for the treatment of respiratory disease. UK patent application No. 6779645.9, granted15th August 2012; Participation on a data safety monitory board or advisory board: Enanta Chair of DSMB, Virtus Respiratory Research Ltd. Shareholder and Board membership. All other authors declare no competing interests.

## Supporting information


Data S1.

Figure S1.

Table S1.

## Data Availability

All datasets generated and analyzed during this study are included in this published article and its Supplementary information files. Publicly available Transcriptome data from bronchial brushings from control individuals and patients with asthma infected in vivo with RV are available under accession number: GSE185658.^32^ Publicly available RNAseq data GSE61141 were downloaded from the NCBI gene expression omnibus.^30^, ^32^ Additional data are available from corresponding author on reasonable request. The PBMCs scRNAseq datasets from patients with severe asthma and control individuals analyzed in the present study, have been deposited in the Sequence Read Archive (SRA) repository under the accession number GSE172495.^49^

## References

[1] Hammad H , Lambrecht BN . The basic immunology of asthma. Cell. 2021;184(6):1469‐1485. 33711259 10.1016/j.cell.2021.02.016

[2] Schiffers C , Hristova M , Habibovic A , et al. The transient receptor potential channel vanilloid 1 is critical in innate airway epithelial responses to protease allergens. Am J Respir Cell Mol Biol. 2020;63(2):198‐208. 32182090 10.1165/rcmb.2019-0170OC PMC7397771

[3] Agache I . Severe asthma phenotypes and endotypes. Semin Immunol. 2019;46:101301. 31466925 10.1016/j.smim.2019.101301

[4] Cardoso‐Vigueros C , von Blumenthal T , Ruckert B , et al. Leukocyte redistribution as immunological biomarker of corticosteroid resistance in severe asthma. Clin Exp Allergy. 2022;52(10):1183‐1194. 35305052 10.1111/cea.14128 PMC9790739

[5] Fahy JV . Type 2 inflammation in asthma—present in most, absent in many. Nat Rev Immunol. 2015;15(1):57‐65. 25534623 10.1038/nri3786 PMC4390063

[6] Lambrecht BN , Hammad H , Fahy JV . The cytokines of asthma. Immunity. 2019;50(4):975‐991. 30995510 10.1016/j.immuni.2019.03.018

[7] Marichal T , Ohata K , Bedoret D , et al. DNA released from dying host cells mediates aluminum adjuvant activity. Nat Med. 2011;17(8):996‐1002. 21765404 10.1038/nm.2403

[8] Decout A , Katz JD , Venkatraman S , Ablasser A . The cGAS‐STING pathway as a therapeutic target in inflammatory diseases. Nat Rev Immunol. 2021;21(9):548‐569. 33833439 10.1038/s41577-021-00524-z PMC8029610

[9] Han Y , Chen L , Liu H , et al. Airway epithelial cGAS is critical for induction of experimental allergic airway inflammation. J Immunol. 2020;204(6):1437‐1447. 32034061 10.4049/jimmunol.1900869

[10] Ozasa K , Temizoz B , Kusakabe T , et al. Cyclic GMP‐AMP triggers asthma in an IL‐33‐dependent manner that is blocked by Amlexanox, a TBK1 inhibitor. Front Immunol. 2019;10:2212. 31616416 10.3389/fimmu.2019.02212 PMC6775192

[11] Raundhal M , Morse C , Khare A , et al. High IFN‐gamma and low SLPI mark severe asthma in mice and humans. J Clin Invest. 2015;125(8):3037‐3050. 26121748 10.1172/JCI80911 PMC4563754

[12] Messaoud‐Nacer Y , Culerier E , Rose S , et al. STING agonist diABZI induces PANoptosis and DNA mediated acute respiratory distress syndrome (ARDS). Cell Death Dis. 2022;13(3):269. 35338116 10.1038/s41419-022-04664-5 PMC8953969

[13] Ahn J , Gutman D , Saijo S , Barber GN . STING manifests self DNA‐dependent inflammatory disease. Proc Natl Acad Sci USA. 2012;109(47):19386‐19391. 23132945 10.1073/pnas.1215006109 PMC3511090

[14] Li XD , Wu J , Gao D , Wang H , Sun L , Chen ZJ . Pivotal roles of cGAS‐cGAMP signaling in antiviral defense and immune adjuvant effects. Science. 2013;341(6152):1390‐1394. 23989956 10.1126/science.1244040 PMC3863637

[15] Jneid B , Bochnakian A , Hoffmann C , et al. Selective STING stimulation in dendritic cells primes antitumor T cell responses. Sci Immunol. 2023;8(79):eabn6612. 36638189 10.1126/sciimmunol.abn6612

[16] Ramanjulu JM , Pesiridis GS , Yang J , et al. Design of amidobenzimidazole STING receptor agonists with systemic activity. Nature. 2018;564(7736):439‐443. 30405246 10.1038/s41586-018-0705-y

[17] Chapman DG , Irvin CG . Mechanisms of airway hyper‐responsiveness in asthma: the past, present and yet to come. Clin Exp Allergy. 2015;45(4):706‐719. 25651937 10.1111/cea.12506 PMC4386586

[18] Lemanske RF Jr , Busse WW . Asthma: clinical expression and molecular mechanisms. J Allergy Clin Immunol. 2010;125(2 Suppl 2):S95‐S102. 20176271 10.1016/j.jaci.2009.10.047 PMC2853245

[19] Wang X , Li Y , Luo D , et al. Lyn regulates mucus secretion and MUC5AC via the STAT6 signaling pathway during allergic airway inflammation. Sci Rep. 2017;7:42675. 28205598 10.1038/srep42675 PMC5312001

[20] Porto BN , Stein RT . Neutrophil extracellular traps in pulmonary diseases: too much of a good thing? Front Immunol. 2016;7:311. 27574522 10.3389/fimmu.2016.00311 PMC4983612

[21] Vorobjeva NV , Pinegin BV . Neutrophil extracellular traps: mechanisms of formation and role in health and disease. Biochemistry. 2014;79(12):1286‐1296. 25716722 10.1134/S0006297914120025

[22] Chen L , Xu J , Deng M , et al. Telmisartan mitigates lipopolysaccharide (LPS)‐induced production of mucin 5AC (MUC5AC) through increasing suppressor of cytokine signaling 1 (SOCS1). Bioengineered. 2021;12(1):3912‐3923. 34281463 10.1080/21655979.2021.1943605 PMC8806622

[23] Zhang CX , Ye SB , Ni JJ , et al. STING signaling remodels the tumor microenvironment by antagonizing myeloid‐derived suppressor cell expansion. Cell Death Differ. 2019;26(11):2314‐2328. 30816302 10.1038/s41418-019-0302-0 PMC6889506

[24] Ray A , Kolls JK . Neutrophilic inflammation in asthma and association with disease severity. Trends Immunol. 2017;38(12):942‐954. 28784414 10.1016/j.it.2017.07.003 PMC5711587

[25] She L , Barrera GD , Yan L , et al. STING activation in alveolar macrophages and group 2 innate lymphoid cells suppresses IL‐33‐driven type 2 immunopathology. JCI Insight. 2021;6(3):e143509. 33400692 10.1172/jci.insight.143509 PMC7934858

[26] Akdis CA . Does the epithelial barrier hypothesis explain the increase in allergy, autoimmunity and other chronic conditions? Nat Rev Immunol. 2021;21(11):739‐751. 33846604 10.1038/s41577-021-00538-7

[27] Tan HT , Hagner S , Ruchti F , et al. Tight junction, mucin, and inflammasome‐related molecules are differentially expressed in eosinophilic, mixed, and neutrophilic experimental asthma in mice. Allergy. 2019;74(2):294‐307. 30267575 10.1111/all.13619

[28] Xiao C , Puddicombe SM , Field S , et al. Defective epithelial barrier function in asthma. J Allergy Clin Immunol. 2011;128(3):549‐556. 21752437 10.1016/j.jaci.2011.05.038

[29] Toussaint M , Jackson DJ , Swieboda D , et al. Host DNA released by NETosis promotes rhinovirus‐induced type‐2 allergic asthma exacerbation. Nat Med. 2017;23(6):681‐691. 28459437 10.1038/nm.4332 PMC5821220

[30] Bai J , Smock SL , Jackson GR Jr , et al. Phenotypic responses of differentiated asthmatic human airway epithelial cultures to rhinovirus. PLoS One. 2015;10(2):e0118286. 25706956 10.1371/journal.pone.0118286 PMC4338293

[31] Jackson DJ , Makrinioti H , Rana BM , et al. IL‐33‐dependent type 2 inflammation during rhinovirus‐induced asthma exacerbations in vivo. Am J Respir Crit Care Med. 2014;190(12):1373‐1382. 25350863 10.1164/rccm.201406-1039OC PMC4299647

[32] Radzikowska U , Eljaszewicz A , Tan G , et al. Rhinovirus‐induced epithelial RIG‐I inflammasome suppresses antiviral immunity and promotes inflammation in asthma and COVID‐19. Nat Commun. 2023;14(1):2329. 37087523 10.1038/s41467-023-37470-4 PMC10122208

[33] Jackson DJ , Sykes A , Mallia P , Johnston SL . Asthma exacerbations: origin, effect, and prevention. J Allergy Clin Immunol. 2011;128(6):1165‐1174. 22133317 10.1016/j.jaci.2011.10.024 PMC7172902

[34] Sykes A , Johnston SL . Etiology of asthma exacerbations. J Allergy Clin Immunol. 2008;122(4):685‐688. 19014758 10.1016/j.jaci.2008.08.017

[35] Motwani M , Pesiridis S , Fitzgerald KA . DNA sensing by the cGAS‐STING pathway in health and disease. Nat Rev Genet. 2019;20(11):657‐674. 31358977 10.1038/s41576-019-0151-1

[36] Corne JM , Marshall C , Smith S , et al. Frequency, severity, and duration of rhinovirus infections in asthmatic and non‐asthmatic individuals: a longitudinal cohort study. Lancet. 2002;359(9309):831‐834. 11897281 10.1016/S0140-6736(02)07953-9

[37] Stirling RG , Chung KF . Severe asthma: definition and mechanisms. Allergy. 2001;56(9):825‐840. 11551247 10.1034/j.1398-9995.2001.00143.x

[38] Holgate ST . Mechanisms of asthma and implications for its prevention and treatment: a personal journey. Allergy, Asthma Immunol Res. 2013;5(6):343‐347. 24179679 10.4168/aair.2013.5.6.343 PMC3810539

[39] Frossing L , Klein DK , Hvidtfeldt M , et al. Distribution of type 2 biomarkers and association with severity, clinical characteristics and comorbidities in the BREATHE real‐life asthma population. ERJ Open Res. 2023;9(2):483. 10.1183/23120541.00483-2022 PMC10026007 36949964

[40] van der Ploeg EK , Krabbendam L , Vroman H , et al. Type‐2 CD8(+) T‐cell formation relies on interleukin‐33 and is linked to asthma exacerbations. Nat Commun. 2023;14(1):5137. 37612281 10.1038/s41467-023-40820-x PMC10447424

[41] Kennedy JL , Pham S , Borish L . Rhinovirus and asthma exacerbations. Immunol Allergy Clin N Am. 2019;39(3):335‐344. 10.1016/j.iac.2019.03.003 PMC6625523 31284924

[42] Ohms M , Moller S , Laskay T . An attempt to polarize human neutrophils toward N1 and N2 phenotypes in vitro. Front Immunol. 2020;11:532. 32411122 10.3389/fimmu.2020.00532 PMC7198726

[43] Garcia‐Navas R , Gajate C , Mollinedo F . Neutrophils drive endoplasmic reticulum stress‐mediated apoptosis in cancer cells through arginase‐1 release. Sci Rep. 2021;11(1):12574. 34131176 10.1038/s41598-021-91947-0 PMC8206108

[44] Murray PJ . Macrophage polarization. Annu Rev Physiol. 2017;79:541‐566. 27813830 10.1146/annurev-physiol-022516-034339

[45] Cloots RHE , Poynter ME , Terwindt E , Lamers WH , Kohler SE . Hypoargininemia exacerbates airway hyperresponsiveness in a mouse model of asthma. Respir Res. 2018;19(1):98. 29792217 10.1186/s12931-018-0809-9 PMC5967058

[46] Litonjua AA , Lasky‐Su J , Schneiter K , et al. ARG1 is a novel bronchodilator response gene: screening and replication in four asthma cohorts. Am J Respir Crit Care Med. 2008;178(7):688‐694. 18617639 10.1164/rccm.200709-1363OC PMC2556451

[47] Maarsingh H , Dekkers BG , Zuidhof AB , et al. Increased arginase activity contributes to airway remodelling in chronic allergic asthma. Eur Respir J. 2011;38(2):318‐328. 21310883 10.1183/09031936.00057710

[48] Xia P , Wang S , Ye B , et al. Sox2 functions as a sequence‐specific DNA sensor in neutrophils to initiate innate immunity against microbial infection. Nat Immunol. 2015;16(4):366‐375. 25729924 10.1038/ni.3117

[49] Chen A , Diaz‐Soto MP , Sanmamed MF , et al. Single‐cell characterization of a model of poly I:C‐stimulated peripheral blood mononuclear cells in severe asthma. Respir Res. 2021;22(1):122. 33902571 10.1186/s12931-021-01709-9 PMC8074196

