# Supplementary material for: STING‐dependent induction of neutrophilic asthma exacerbation in response to house dust mite
Source: Allergy. 2024 Oct 28;80(3):715–37. doi: 10.1111/all.16369 (PMC11891437; doi:10.1111/all.16369)
Supplement: Supplementary file 1 — Data S1. [file ALL-80-715-s001.pdf]

Online supplemental materials

STING-dependent induction of neutrophilic asthma exacerbation in response to house dust mite.

Yasmine Messaoud-Nacer, PhD[1], Elodie Culerier, MSc[1], Stéphanie Rose, MSc[1], Isabelle Maillet, MSc[1], Rania Boussad MSc[1], Chloé Veront MSc[1], Florence Savigny MSc[1], Bernard Malissen, PhD[2], Urszula Radzikowska, PhD[3,4], Milena Sokolowska, MD[3,4], Gabriel VL da Silva, PhD[5], Michael R Edwards, PhD[6], David J Jackson, MD, PhD[6], Sebastian L Johnston, MD, PhD[6], Bernhard Ryffel, MD, PhD[1], Valerie F. Quesniaux, PhD[1], and Dieudonnée Togbe, PhD[1*]

Methods

Airway resistance measurement

Mice were anesthetized with intraperitoneal (i.p.) injection of a solution containing ketamine (100 mg/kg, Merial) and xylasine (10 mg/kg, Bayer), paralyzed using D-tubocurarine (0.125%, Sigma), and intubated with an 18-gauge catheter. Respiratory frequency was set at 140 breaths per min with a tidal volume of 0.2 ml and a positive end-expiratory pressure of 2 ml H2O. Increasing concentrations of aerosolized methacholine (25, 50, 100 and 200 mg/mL) were administered. Airway resistance was measured using a ventilator (Elan Series Mouse RC Site, Buxco, Electronics) and BioSystem X.A. software (Buxco Electronics). Baseline resistance was restored before administering the subsequent doses of methacholine.

Immunoblots

Lung tissues were homogenized in T-PER™ buffer supplemented with a cocktail containing protease and phosphatase inhibitors (halt™, ThermoFisher). Total protein was extracted and quantified by using Pierce™ BCA Protein Assay Kit (ThermoFisher®). Total protein (40 µg) was treated with NuPAGE™ LDS sample buffer and sample reducing agent (ThermoFisher®), and heated 10 min at 70°C. Samples were resolved on 4-12% polyacrylamide gel (Bolt™ Mini protein gel, ThermoFisher) and run at 160V for 45min using the Mini gel Tank (ThermoFisher®). Total protein was electroblotted to 0.2 µm nitrocellulose membrane (Amersham™, UK) using a Trans-Blot SD Transfer System (Bio-Rad, California) at 100V for 45 min. Successful protein transfer was confirmed by using Ponceau S staining. Membranes were blocked with 5% nonfat milk (Cell signaling, Massachusetts) in 1X TBS-T (20 mMTris Base, 150 mM sodium chloride, and 0.05% Tween-20 pH 7.6) for 1h at room temperature.

Full membranes or portions of membranes were incubated overnight using primary antibodies from rabbit anti-phospho-STAT6 (#56554 1/500; Cell signaling), anti-STAT6 (#5397 1/500;

Cell signaling), anti-histone H3 (#ab281584 1/500; Abcam, UK), anti-phospho-γH2AX (#9718 1/500; Cell signaling), anti-γH2AX (#7631 1/500; Cell signaling), anti-phospho-STING (#72971 1/500; Cell signaling), anti-STING (#13647 1/500; Cell signaling), anti-phospho-TBK1 (#5483 1/500; Cell signaling), anti-TBK1 (#3504 1/500; Cell signaling), anti-phospho-IRF3 (#4947 1/500; Cell signaling), IRF3 (#4302 1/500; Cell signaling), anti- cGAS (#31659 1/500; Cell signaling), anti-DDX41 (#15076 1/500; Cell signaling), anti-IFI204 (#ab228512 1/500; Abcam), anti-AIM2 (#63660 1/500; Cell signaling), anti-NLRP3 (#15101 1/500; Cell signaling), anti-caspase-11/cleaved-caspase-11 (#NB120-10454 1/500; Novus), anti-IL-1β (#12426 1/500; Cell signaling), anti-IL-18 (#57058 1/500; Cell signaling), anti-cleaved caspase-3 (#9661 1/500), anti-caspase-3 (#9662 1/500; Cell signaling), anti-MLKL (#37705 1/500; Cell signaling), anti-cleaved GSDMD (#10137 1/500; Cell signaling), anti-GSDMD (#ab219800 1/500; Abcam), mouse anti-ZBP1 (#SC-271483 1/300; Santa Cruz, Texas), anti-caspase-8 (#4927 1/500; Cell signaling), anti-RIPK3 (#95702, 1/500, Cell signaling), anti IL-25 (# PA5-100835, 1/500, Invitrogen) and anti-actinβ (#A3854 1/10000; Sigma-Aldrich, Massachusetts). Membranes were washed in TBS-T three times for 10 min each at room temperature, and then incubated with goat anti-rabbit-IgG-HRP-conjugate (#7074 1/2000; Cell signaling) or horse anti-mouse-IgG-HRP-conjugate (#7076 1/2000; Cell signaling) diluted in 5% non-fat milk in TBS-T for 1h at RT. The membranes were washed three times in TBS-T. Protein bands were visualized following exposure of the membrane to Amersham ECL™ prime substrate solution (Cytiva, Massachusetts) on film iBright 1500 (Invitrogen, Massachusetts).

Immunofluorescence staining of lung and BAL cells

Lungs were fixed with 4% formalin for 72h, embedded in paraffin and sectioned at 3 μm. Lung sections were dewaxed and rehydrated, then heated 20 min at 80°C in citrate buffer 10 mM pH=6 for antigen retrieval (unmasking step). Lung sections were permeabilized in PBS 0.5% triton X-100, blocked with 5% FCS for 1h at RT and then incubated overnight with primary

goat antibodies to MPO (1:40, R&D systems, Minneapolis) or rabbit antibodies to ZO-1 (1:200, Abcam, UK). After washing, the sections were incubated with donkey anti-goat IgG secondary antibodies conjugated with AlexaFluor488 (1:200, Invitrogen) and goat anti-rabbit IgG secondary antibodies conjugated with AlexaFluor 568 (1:500, Invitrogen, Massachusetts) in 1% FCS. Following washing, lung sections were counterstained with DAPI (1:1000) and mounted onto microscope slides (Fluoromount-G, Invitrogen). Cytospin slides were fixed in 4% PFA. Cells were washed 3 times in TBS, incubated 15 min in TBS-0.3% Triton X-100, then washed 3 times in TBS, blocked in TBS-10% FCS for 45 min and incubated overnight with primary antibodies to MPO (1:40, R&D systems), and to histone H3-citrulline (1:100, Abcam) for NETs visualization. For PANoptosome visualization slides were incubated with primary antibodies from mouse anti-ZBP1 (1:100, Santa Cruz), rat anti-Caspase 8 (1: 100, Enzo Lifesciences, France), goat anti-ASC (1:50, Abcam), and mouse anti-RIPK3 (B-2) conjugated to Alexa Fluor® 647 (1:30, Santa Cruz Biotechnology). After washing, slides were incubated as described above for NETs formation detection. For the analysis of PANoptosome components, slides were incubated with donkey anti-goat IgG secondary antibodies conjugated with AlexaFluor488 (1:200, Invitrogen) and goat anti-rat secondary antibodies Alexa Fluor 546. Slides were counterstained using DAPI for 10 min. For NETs visualization, lung tissue and cells were observed using a Leica DM 6000B microscope (HAMAMATSU ORCA-Fusion C14440 Camera, Japan), images were acquired using MetaMorph® software and were treated using ImageJ software. For PANoptosome visualization, cells were observed using a Zeiss LSM 980 confocal microscope coupled with a Zeiss Airyscan 2 device (Carl Zeiss Co. Ltd., Jena, Germany). Images were acquired using Zeiss LSM Image Browser (Carl Zeiss Co. Ltd., Jena, Germany).

Flow cytometry

Bronchoalveolar lavage cells were counted and plated on a 96 well plate for extracellular staining. Different subsets of lung infiltrating cells or resident cells were detected by flow cytometry in BAL cell suspensions using a mix of the following fluorochrome-conjugated antibodies against mouse NOS2 PE-Cy7 (1/100 eBioscience), Arginase1-PE (1/100 eBioscience), Siglec F-PE-Cf59 (1/300, BD), CD45-PerCP (1/200, Biolegend), MPO-FITC (1/200, ThermoFisher), CD11b-BV786 (1/200, Fisher scientific), LY-6G-BV605 (1/100, BD), F4/80- BV421 (1/200, Biolegend), CD45-APC (1/200, Biolegend), F4/80-PE/Cy7 (1/200, Biolegend), Siglec F-PE-Cf59 (1/300, BD), CD3e-PE (1/100, BD), CD193 (CCR3)-PercPVio700 (1/50, Miltenyi Biotec), CD182 (CXCR2)-R718 (1/100, BD), CD11b-BV786 (1/200, Fisher scientific), CD45R/B220-BV711 (1/200, BD), CD11c-BV605 (1/200, BD), Ly-6G-BV510 (1/100, BD), IA/IE-eF450 (1/200, Fisher scientific), CD184 (CXCR4)-PE (1/100, Biolegend) and Fc Block (1/200) to avoid nonspecific binding. All staining reactions were performed at RT for 20 to 30 min. Flow cytometry analyses were performed on LSR Fortessa X-20 flow cytometer (Becton Dickinson, New Jersey). Gating strategy was set up according to FMO contros for all antibodies. Analysis and graphical output were performed using FlowJo™ software (Tree Star, Ashland, OR).

Lactate dehydrogenase (LDH) Measurement

Cytotoxicity was determined by quantifying lactate dehydrogenase (LDH) released in the supernatant of human epithelial cells using LDH-Glo^{TM}- Cytotoxicity assay kit (Promega) according to the manufacturer's instructions.

Quantification of mRNA expression by RT-qPCR analysis

Total mRNA was extracted using TRIzol (TRI-Reagent, Sigma-Aldrich, Germany) and reverse transcribed in cDNA with GoScript™ Reverse Transcription kit (Promega, Wisconsin). Genes mRNA expression were analyzed using GoTaq®qPCR Master Mix (Promega). All primer sequences used were from Qiagen: *Tmem173* (#QT00261590), *Mb21d1* (#QT00131929), *Ifi204* (#QT01753535), *Aim2* (#QT00266819), *Serpinb2* (#QT01052345), *Clac1* (#QT00164290); *Spdef* (#QT0010719), *Socs1* (#QT01059268), *Hif1-α* (#QT01039542), *Osm (#QT00263193)*, *Tlr-9 (QT01043049)*, *IL-4 (*#QT00160678*)*, *IL-5(*#QT00099715*)*, *IL-13 (QT00099554). Muc5ac (Forward:* CAGCCGAGAGGAGGGTTTGATCT. Reverse: AGTCTCTCTCCGCTCCTCTCAAT*), MUC5AC (*#QT00088991)*,* RNA expression was normalized to *Rn18s* expression (Qiagen, Maryland). Data were analyzed using the comparative analysis of relative expression by $^{\Delta\Delta}$Ct methods.

Histology

Lung left lobe was removed and fixed in 4% formalin, embedded in paraffin, sectioned at 3 µm, stained with periodic acid-Schiff (PAS) and blindly scored by an anatomo-pathologist. Semi quantitative scoring (0-5) of Goblet cell and airway inflammation including epithelial injury, and peribronchial infiltrates severity were performed.

Double-stranded DNA Measurement in BALF

dsDNA was measured in the acellular fraction of the BALF using Quant-iT PicoGreen (Invitrogen, Massachusetts) according to the manufacturer's protocol.

Measurement of cytokine levels

MPO, IL-6, TNFα, CCL11, CCL24, CXCL1, and CXCL10 concentrations in BALF and lung homogenate were measured by ELISA (R&D System, Minneapolis). IFNα, IFNβ, IFN-γ, IL-4, IL-5 and IL-13, levels in BALF and lung homogenate, and IFNα, IFNβ, IFN-γ, CXCL-8 CXCL-

10, IL-6, TFN-α levels in cell culture supernatant were quantified by multiplex immunoassay according to manufacturers' instructions (ProcartaPlex, Life Technologies, Massachusetts). Data were acquired on Luminex equipment (MagPix, BioRad, California) and analyzed using Bioplex Manager software (BioRad).

MUC5AC measurements

MUC5AC levels were quantified in the acellular fraction of the BALF and lung homogenate using the Mouse MUC5AC (Mucin-5 subtype AC) ELISA kit (Elabscience®, Texas) according to the manufacturer's instructions.

Dose response study of STING agonists

Mice were anesthetized with 2% Isoflurane (ISO-VET, Netherlands) and sensitized with HDM on day 0 and 7 (25 µg/mouse, i.n.) and challenged with HDM intranasally on 3 consecutive days (10 µg/mouse, i.n. on day 14-16), in the absence or together with cGAMP (at 1, 3 or 10 µg/mouse, i.t.), diABZI compound 3 (at 0.1 or 1 µg/mouse, i.t) or Poly(I:C) (at 60 or 200 µg/mouse, i.t). Mice were analyzed on day 17, 24 hours after the last HDM instillation.

Bronchoalveolar lavage (BAL) was performed 24h after the last challenge by flushing lung tissue four times with 0.5 mL of cold NaCl 0.9% via tracheal intubation with a cannula. BALF was collected, cells counted and cytospins performed. The supernatant of the first lavage was collected after centrifugation and stored at -80°C for dsDNA and mediators quantification. The left lung lobe was harvested for histology, the post caval lung for RNA extraction and qPCR analysis and the right lobes for Western blot analysis and cytokine measurement. Protein extravasation in the BALF was measured by Pierce™ BCA Protein Assay (ThermoFisher®, Massachusetts).

Single cell RNA sequencing analysis

We re-analyzed single-cell transcriptomic data (GSE172495) from Human Asthma PBMC and control PBMC [1]. The dataset was downloaded and the RDS file was imported into R [2] environment version v4.3.0 and Seurat v4.3.0 [3] by filtering genes expressed in at least 200 cells and cells expressing at least 3 genes. For the pre-processing step, outlier cells were filtered out based on three metrics (nCount_RNA < 4000, nFeature_RNA >= 200 & nFeature_RNA < 2500 and mitochondrial percentage expression <5). The top 2,000 variable genes were then identified using the 'vst' method using the *FindVariableFeatures* function. Percent of mitochondrial genes was regressed out in the scaling step, and Principal Component Analysis (PCA) was performed using the top 2,000 variable genes and the top 50 PCs were selected for dimension reduction by Uniform Manifold Approximation and Projection (UMAP). Clusters were identified using the authors annotation. Then, differential gene expression analysis was performed using *FindAllMarkers* function in Seurat with default parameters to obtain a list of significant gene markers for each cluster of cells. Visualization of genes illustrating expression levels was performed using R/Seurat commands (DimPlot, FeaturePlot and DotPlot) using ggplot2 [4] and scCustomize [5] R packages.

In vitro RV-A16 infection of human bronchial epithelial cells

Human bronchial epithelial cells (HBECs) obtained from six healthy individuals and six patients diagnosed with asthma were infected with RV-A16 at an MOI of 10 for 24h and subjected to sequencing using the Illumina HiSeq 2000 platform as described [6, 7]. Transcriptome data were processed with the workflow available here [https://github.com/uzh/ezRun using the edgeR R package [7]. Presented gene sets were curated from GSEA and MSigDB Database (Broad Institute, Massachusetts Institute of Technology, and Reagent of the University of California, USA). Full sets of analyzed genes are described in Supplementary Table 1. Asterisks demonstrate significantly changed genes with threshold p<0.05. Heatmaps display normalized gene expression across the gene in the groups (row normalization).

Transcriptome analyses of experimental in vivo RV-A16 infection in humans

Transcriptomics of bronchial brushings obtained from control individuals (n=7) and patients with asthma (n=17) 14 days before and 4 days after experimental RV-A16 infection in vivo [6-8]were collected and analyzed as previously described [7] with Affymetrix HuGene 1.0 array and Transcriptome Analysis Console v4.0 (Santa Clara, United States). Data was analyzed by the Bioconductor microarray analysis workflow available here [https://www.bioconductor.org/packages/release/workflows/vignettes/arrays/inst/doc/arrays.html]. Presented gene sets were curated from GSEA and MSigDB Database (Broad Institute, Massachusetts Institute of Technology, and Reagent of the University of California, USA). Full sets of analyzed genes are described in Supplementary Table 2. Asterisks demonstrate significantly changed genes with threshold p<0.05. Heatmaps display normalized gene expression across the gene in the groups (row normalization).

Supplemental Figure legends

Suppl E1: Synthetic STING agonist diABZI triggers neutrophilic asthma exacerbation in HDM sensitized mice, induces DNA damages and upregulation of DNA sensors.

Mice sensitized with HDM on day 0 and 7 (25 µg, i.n.) and challenged with HDM on day 14-16 (10 µg/day, i.n.) received diABZI (at 0.1 or 1 µg/mouse, i.t.) or NaCL and analyzed on day 17. **(A)** Neutrophils and **(B)** Eosinophils counts in BAL. **(C, D)** MPO concentration in BALF and lung. **(E)** Concentration of extracellular dsDNA in the acellular fraction of the BALF. **(F)** IL-4, **(G)** IL-5 and (**H**) IFN-γ concentrations in BALF quantified by Luminex immunoassay. **(I)** Lung tissue histology PAS staining Bars, left panel: 2.5mm, right panel: 250µm, with pathology scoring of **(J)** goblet cells **(K)** Peribronchial infiltrates **(L)** Epithelial injury. **(M)** *Muc5ac* transcripts measured by real-time PCR. **(N)** HDM-specific IgE and **(O)** total IgE concentrations in serum. Data were presented as mean ± SEM with n = 4-6 mice per group. Each point represents an individual mouse. *$p < 0.05$, **$p < 0.01$ (Nonparametric Kruskal–Wallis with Dunn's post-test).

Suppl E2: Endogenous STING agonist 2'3'-cGAMP promotes lung neutrophilia, NETs formation, DNA damage and exacerbate the inflammatory response to House dust mite.

Mice sensitized with HDM on day 0 and 7 (25 µg, i.n.) were challenged intranasally with HDM on 3 consecutive days (10 µg, i.n. on day 14-16), in the absence or together with cGAMP (at 1, 3 or 10 µg/mouse, i.t.), and parameters analyzed on day 17. **(A)** Neutrophils and **(B)** Eosinophils counts in BAL. **(C, D)** MPO concentration in BALF and lung. **(E)** Concentration of extracellular dsDNA in the acellular fraction of the BALF. **(F)** IL-4, **(G)** IL-5 and (**H**) IL-13 and **(I)** IFN-γ concentrations in BALF quantified by Luminex immunoassay. **(J)** Lung tissue histology PAS staining Bars, left panel: 2.5mm, right panel: 250µm, with pathology scoring of **(K)** goblet cells **(L)** Peribronchial infiltrates **(M)** Epithelial injury. **(N)** *Muc5ac* transcripts

measured by real-time PCR. Data were presented as mean ± SEM with n = 6-15 mice per group. Each point represents an individual mouse. *p < 0.05, **p < 0.01, ***p < 0.001, ****p < 0.0001 (Nonparametric Kruskal–Wallis with Dunn's post-test).

Suppl E3: Effect of Poly(I:C) on allergic asthma response to house dust mite.

Mice sensitized with HDM on day 0 and 7 (25 µg, i.n.) were challenged with HDM on day 14-16 (10 µg/day, i.n.) in the absence or together with Poly(I:C) at 60 or 200 µg/mouse, i.t. and analyzed on day 17. **(A)** Neutrophils and **(B)** Eosinophils counts in BAL. **(C, D)** MPO concentration in BALF and lung. **(E)** Concentration of extracellular dsDNA in the acellular fraction of the BALF. **(F)** IL-4, **(G)** IL-5 and (**H**) IL-13 and **(I)** IFN-γ concentrations in BALF quantified by Luminex immunoassay. **(J)** Lung tissue histology PAS staining Bars, left panel: 2.5mm, right panel: 250µm, with pathology scoring of **(K)** goblet cells **(L)** Peribronchial infiltrates **(M)** Epithelial injury. Data were presented as mean ± SEM with n = 8 mice per group. Each point represents an individual mouse. *p < 0.05, **p < 0.01, ***p < 0.001, ****p < 0.0001 (Nonparametric Kruskal–Wallis with Dunn's post-test).

Suppl E4 related to Fig.1: STING agonists diABZI and cGAMP induce neutrophilic asthma exacerbation

(A), Total cells **(B)** macrophages and **(C)** lymphocytes counts in BAL. **(D)** Gating strategy of NOS2/ARG1 staining of pre-gated singlets (SSC-A/SSC-H), $CD45^+$ (leukocytes) and $CD45^+CD11b^+Ly6G^+F4/80^-SiglecF^-$ (neutrophils). **(E)** Concentration of proteins in BALF. **(F-K)** Th2 cytokines **(F, I)** IL-4, **(G, J)** IL-5 and **(H, K)** IL-13 in BALF and lung determined by multiplex immunoassay. **(L, M)** Eotaxine1 (CCL11) in BALF and lung measured by ELISA. **(N, O)** IL-6 concentrations in BALF and lung determined by ELISA. **(P)** TNF-α concentration in BALF determined by ELISA. **(Q-S)** Epithelial-derived alarmins, (**Q**) IL-33, (**R**) TSLP, (**S**)

Immunoblot of IL-25 protein and quantification. (**T, U**) Th2 attracting chemokines (**T**) CCL17 and (**U**) CCL22 in lung. **(V)** Uncropped immunoblots of p STAT6, STAT6, Actinβ. Data are presented as mean ± SEM with n = 8 mice per group. Each point represents an individual mouse. *$p < 0.05$, **$p < 0.01$ (Nonparametric Kruskal–Wallis with Dunn's post-test).

Suppl E5 related to Fig.2: HDM-induced lung inflammation is exacerbated by airway dsDNA released after NETosis and PANoptosis.

(A-D) Uncropped immunoblots of Cit-H3, pγH2AX, γH2AX, pSTING, STING, pTBK1, TBK1, cGAS, DDX41, IfI204, AIM2, NLRP3, CASP-11, cleaved CASP-11, cleaved IL-1β, IL-18, CASP-3, cleaved CASP-3, GSDMD, cleaved GSDMD, MLKL, ZBP1, RIPK3, CASP-8 and Actinβ. **(E)** Confocal microscopy showing Caspase-8 (green), ASC (red), RIPK3 (far-red/turquoise blue), and DNA dye DAPI (cyan) in granulocytes from BAL of mice treated by HDM challenged or not with diABZI 1 µg, cGAMP 10 µg, or Poly(I:C) 200 µg.

Suppl E6 related to Fig.3: Asthma exacerbation induced by the STING agonist diABZI is glucocorticoid-resistant

(A-D) CXCL1 in BALF and lung, CXCL10 and CXCL11concentrations in BALF. **(E, F)** TNF-α and IL-6 in lung. **(G, H)** CCL17 and CCL22 concentrations in lung. **(E)** Immunoblot of IL-25 protein and quantification. **(K-M)** Uncropped Immunoblots of IL-25 and Actinβ. Data were presented as mean ± SEM with n = 8 mice per group. Each point represents an individual mouse. *$p < 0.05$, **$p < 0.01$ and ****$p < 0.0001$ (Nonparametric Kruskal–Wallis with Dunn's post-test).

Suppl E7 related to Fig.4: STING specificity of diABZI induced asthma exacerbation

(A) total cells, **(B)** eosinophils, **(C)** macrophages and **(D)** lymphocytes counts in BAL. **(E-G)**: Th2 cytokines **(E)** IL-4, **(F)** IL-5 and **(G)** IL-13 concentration in BALF measured by multiplex immunoassay. **(H)** *Il-4* and **(I)** *Il-5* transcripts in lung analyzed by real time qPCR. **(J-L)** Eotaxins **(J, K)** CCL11 concentrations in BALF and lung, **(L)** CCL24 concentration in BALF determined by ELISA. Flow cytometry analysis of **(O)** Neutrophils in BAL **(P)** Neutrophils expressing $CXCR2^+$ in BAL **(Q)** Neutrophils expressing $CXCR4^+$ in BAL **(R)** Gating strategy of CXCR2 and CXCR4 staining of pre-gated singlets (SSC-A/SSC-H), $CD45^+$ $CD11c^-$ and $CD45^+CD11c^-CD3\text{-}B220^-CD11^+Ly6G^{high}$ (neutrophils). Data are presented as mean ± SEM with n = 6 ~ 8 mice per group. Each point represents an individual mouse. $^*p < 0.05$, $^{**}p < 0.01$, $^{***}p < 0.001$, $^{****}p < 0.0001$ (Nonparametric Kruskal–Wallis with Dunn's post-test).

Suppl E8 related to Fig.5: Generation, identification and induction of allergic lung inflammation in myeloid-specific STING-deficient ($STING\text{-}OST^{fl}LysM^{Cre/+}$) mice.

(A) Mice conditionally containing a loxP sequence flanking the third exon of STING gene ($STING\text{-}OST^{fl/fl}$) were crossed with Lysozyme M^{Cre} mice, which expressed Cre recombinase downstream of the lysozyme LysM promoter ($LysM^{Cre}$). **(B)** Mice were genotyped using PCR analysis of DNA obtained from tail snip. STING-deficient ($STING\text{-}OST^{fl}LysM^{Cre/+}$) mice have a 722-bp product for the loxP-targeted allele and a 700-bp product for the $LysM^{Cre}$ allele. $STING\text{-}OST^{fl/fl}$ mice have a 722-bp product for the loxP-targeted allele and a 501-bp product for the WT allele. **(C)** STING protein expression of STING-OST in BMDMs by immunoblot. **(D)** Flow cytometry analysis of OST producing cells in $STING\text{-}OST^{fl}LysM^{Cre/+}$ BMDMs. All genotypes were generated on C57BL6/N background. Data are presented from one experiment (n = 2, B–D). **(E)** total cells count in BAL **(F)** Proteins level in BALF. **(G)** Gating strategy of Neutrophils ($CD45^+CD11c^-CD3^-B220^-CD11b^{high}$ $Ly6G^{high}$) and Eosinophils ($CD45^+CD11c^-$

$CD3^-$ $B220^-$ $SiglecF^+$ $IAIE^-$ $CD11b^+$ $Ly6G^-$) cells in BAL pre-gated on singlets (SSC-A/SSC-H), $CD45^+$ $CD11c^-$ and non-lymphoid cells $CD3^-$ $B220^-$. Data are presented as individual mouse.

Suppl E9 related to Fig.6: DiABZI disrupted the integrity of epithelial barrier and caused epithelial DNA damage

(**A**) Uncropped immunoblots of pSTAT6, STAT6, pSTING, STING pγH2AX, γH2AX, pTBK1, TBK1, pIRF3, IRF3, p MLKL, MLKL, pγH2AX and Actinβ.

(**B**) Immunofluorescence images quantification of ZO-1 protein.

(**C**) Uncropped immunoblots of pγH2AX and Actinβ of cells from healthy controls and patient with asthma.

Suppl E10 related to Fig.7: Single-cell RNAseq of PBMCs from healthy individuals or patients with severe asthma

(**A**) PBMC cells isolated from whole blood of 5 patients with severe asthma A (n=6099) and 3 healthy individuals (n=3315) in basal condition, and subjected to gene profiling using scRNA-Seq as described in the original study [1]. (**B-C**) Dot-plots showing the expression of (**B**) STING pathway-related genes and (**C**) PANoptosis-related genes. Publicly available data under accession number: GSE172495.

Suppl E11 related to Fig.7: Upregulation of DNA sensing and PANoptosis pathways upon rhinovirus infection in patients with asthma, as compared to non-infected individuals

(**A**) Human bronchial epithelial cells (HBECs) from patients with asthma (n = 6) and healthy controls (n=6), were infected with RV-A16 for 24h, and subjected to transcriptome analysis. (**B-D**) Heatmaps presented together with the corresponding log2 fold change (FC) expression changes (black bars) of (**B**) STING pathway-related genes, (**C**) Tight junction genes set, (**D**) PANoptosis-related genes, before in vitro RV-A16 infection in controls compared to HBEC from patients with asthma. (**E-F**) Heatmaps of mucus pathway (**E**) after and (**F**) before *in vitro* RV-A16 infection in controls compared to HBEC from patients with asthma.

(**G**) Experimental in vivo RV infection in patients with asthma (n=17) and healthy individuals (n=7): Transcriptomic analysis of bronchial brushings 14 days before and 4 days after the infection Heatmaps presented together with the corresponding log2 fold change (FC) expression changes (black bars) of (**H**) Tight junction genes set, (**I**) PANoptosis-related genes, 14 days before in vivo RV-A16 infection in controls compared to patients with asthma.

Data was analyzed by the Bioconductor microarray analysis workflow available here [https://www.bioconductor.org/packages/release/workflows/vignettes/arrays/inst/doc/arrays.html].

All Heatmaps displays normalized gene expression across the groups (row normalization). Asterisks demonstrate significantly changed genes with threshold $p<0.05$. p-value: *<0.05. Publicly available data under accession number: GSE185658 and GSE61141.

References

1. Chen A, Diaz-Soto MP, Sanmamed MF, Adams T, Schupp JC, Gupta A, et al. Single-cell characterization of a model of poly I:C-stimulated peripheral blood mononuclear cells in severe asthma. Respir Res 2021; 22:122.
2. Team RC. R: A Language and Environment for Statistical Computing_. R Foundation for Statistical Computing,. https://www.R-project.org/, 2023.
3. Hao Y, Hao S, Andersen-Nissen E, Mauck WM, 3rd, Zheng S, Butler A, et al. Integrated analysis of multimodal single-cell data. Cell 2021; 184:3573-87 e29.
4. Wickham H. ggplot2: Elegant Graphics for Data Analysis. ; 2016.
5. Marsh S. scCustomize: Custom Visualizations & Functions for Streamlined Analyses of Single Cell Sequencing_. R package version 1.1.1. https://CRAN.R-project.org/package=scCustomize, 2023.
6. Jackson DJ, Makrinioti H, Rana BM, Shamji BW, Trujillo-Torralbo MB, Footitt J, et al. IL-33-dependent type 2 inflammation during rhinovirus-induced asthma exacerbations in vivo. Am J Respir Crit Care Med 2014; 190:1373-82.
7. Radzikowska U, Eljaszewicz A, Tan G, Stocker N, Heider A, Westermann P, et al. Rhinovirus-induced epithelial RIG-I inflammasome suppresses antiviral immunity and promotes inflammation in asthma and COVID-19. Nat Commun 2023; 14:2329.
8. Farne H, Lin L, Jackson DJ, Rattray M, Simpson A, Custovic A, et al. In vivo bronchial epithelial interferon responses are augmented in asthma on day 4 following experimental rhinovirus infection. Thorax 2022; 77:929-32.
