# Supplementary material for: STING‐dependent induction of neutrophilic asthma exacerbation in response to house dust mite
Source: Allergy. 2024 Oct 28;80(3):715–37. doi: 10.1111/all.16369 (PMC11891437; doi:10.1111/all.16369)

Suppl Fig.E1

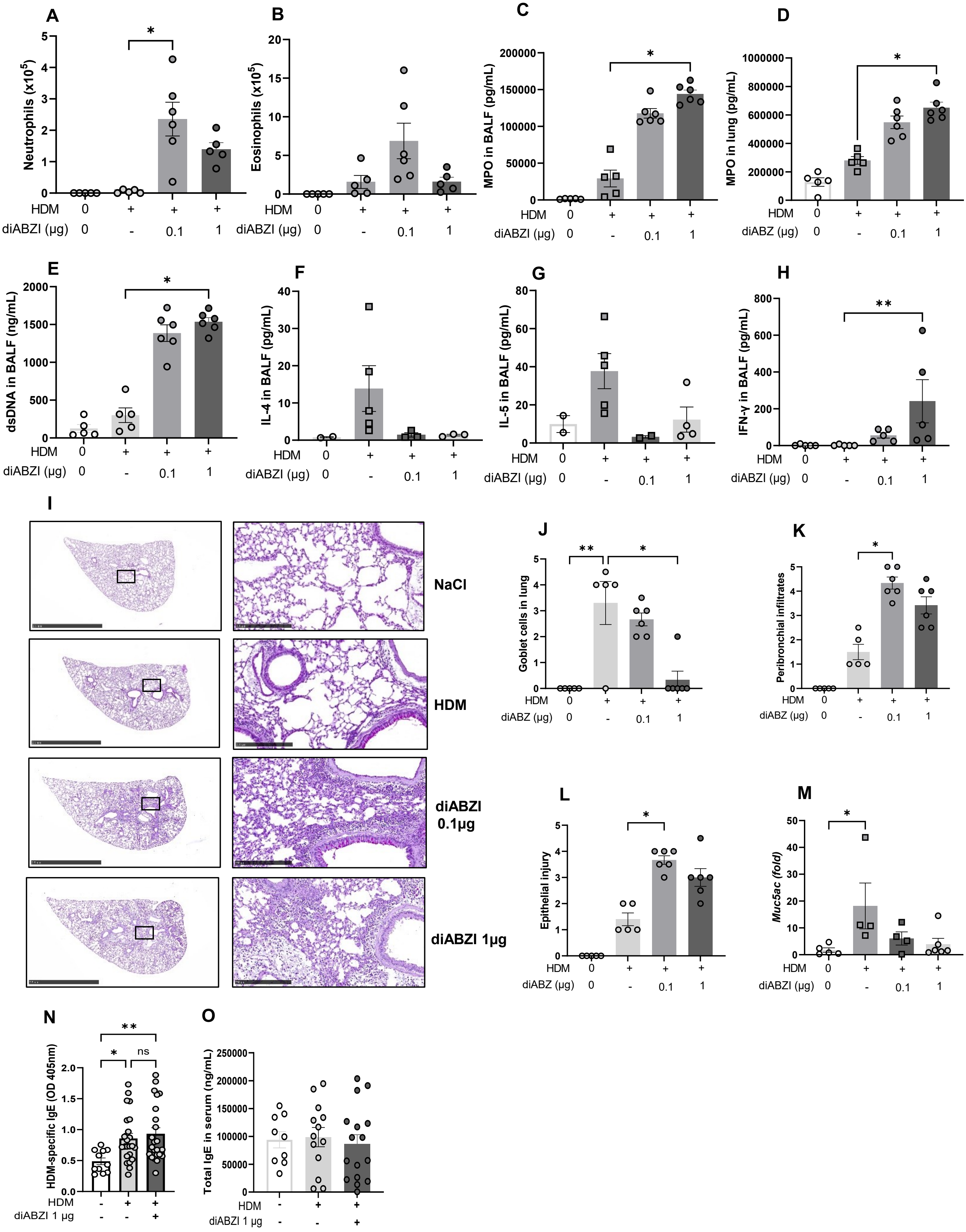

A
Neutrophils ($x10^5$)
B
Eosinophils ($x10^5$)
C
MPO in BALF (pg/mL)
D
MPO in lung (pg/mL)
E
dsDNA in BALF (ng/mL)
F
IL-4 in BALF (pg/mL)
G
IL-5 in BALF (pg/mL)
H
IFN-γ in BALF (pg/mL)
I
NaCl
HDM
diABZI 0.1μg
diABZI 1μg
J
Goblet cells in lung
K
Peribronchial infiltrates
L
Epithelial injury
M
Muc5ac (fold)
N
HDM-specific IgE (OD 405nm)
O
Total IgE in serum (ng/mL)
HDM
diABZI (μg)
diABZ (μg)
diABZI 1 μg
ns

Suppl Fig.E2

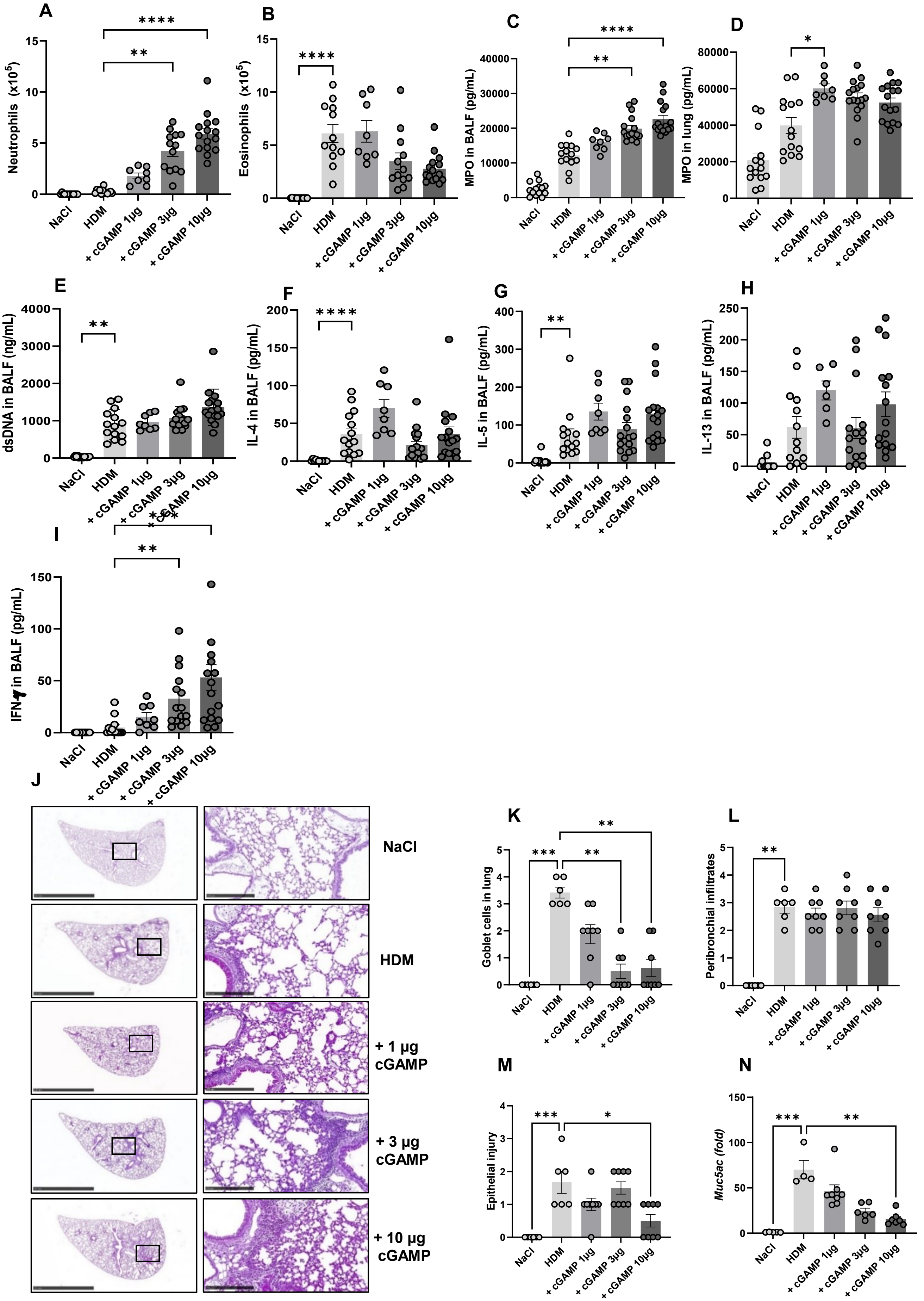
A
B
C
D
E
F
G
H
I
J
K
L
M
N
Neutrophils (x10^5)
Eosinophils (x10^5)
MPO in BALF (pg/mL)
MPO in lung (pg/mL)
dsDNA in BALF (ng/mL)
IL-4 in BALF (pg/mL)
IL-5 in BALF (pg/mL)
IL-13 in BALF (pg/mL)
IFN-γ in BALF (pg/mL)
Goblet cells in lung
Peribronchial infiltrates
Epithelial injury
Muc5ac (fold)
NaCl
HDM
+ cGAMP 1µg
+ cGAMP 3µg
+ cGAMP 10µg
+ 1 µg cGAMP
+ 3 µg cGAMP
+ 10 µg cGAMP

Suppl Fig.E3

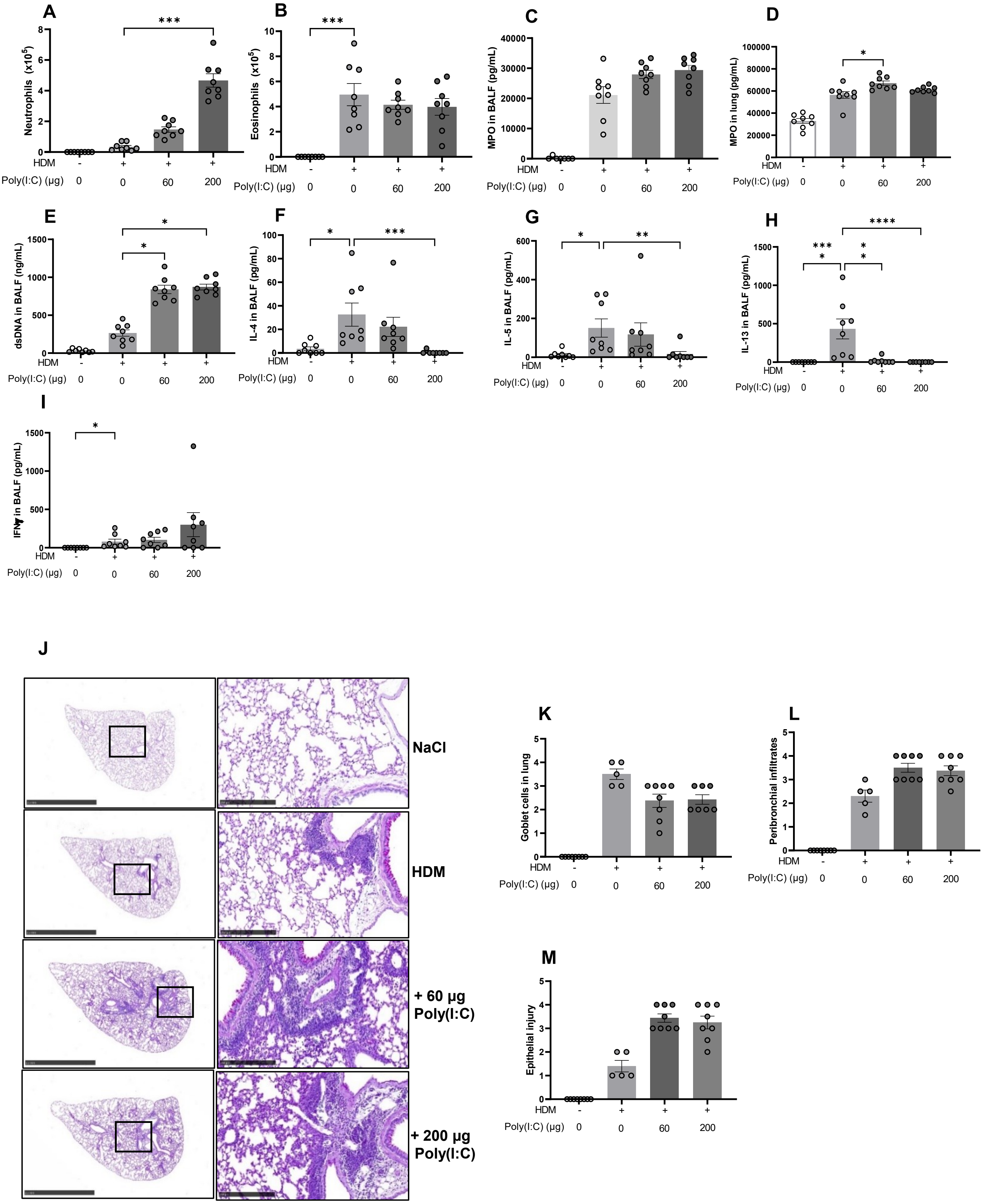

A
Neutrophils (x10^5)
B
Eosinophils (x10^5)
C
MPO in BALF (pg/mL)
D
MPO in lung (pg/mL)
E
dsDNA in BALF (ng/mL)
F
IL-4 in BALF (pg/mL)
G
IL-5 in BALF (pg/mL)
H
IL-13 in BALF (pg/mL)
I
IFNγ in BALF (pg/mL)
HDM - + + +
Poly(I:C) (µg) 0 0 60 200
J
NaCl
HDM
+ 60 µg Poly(I:C)
+ 200 µg Poly(I:C)
K
Goblet cells in lung
L
Peribronchial infiltrates
M
Epithelial injury

Suppl Fig.E4 related to Fig.1

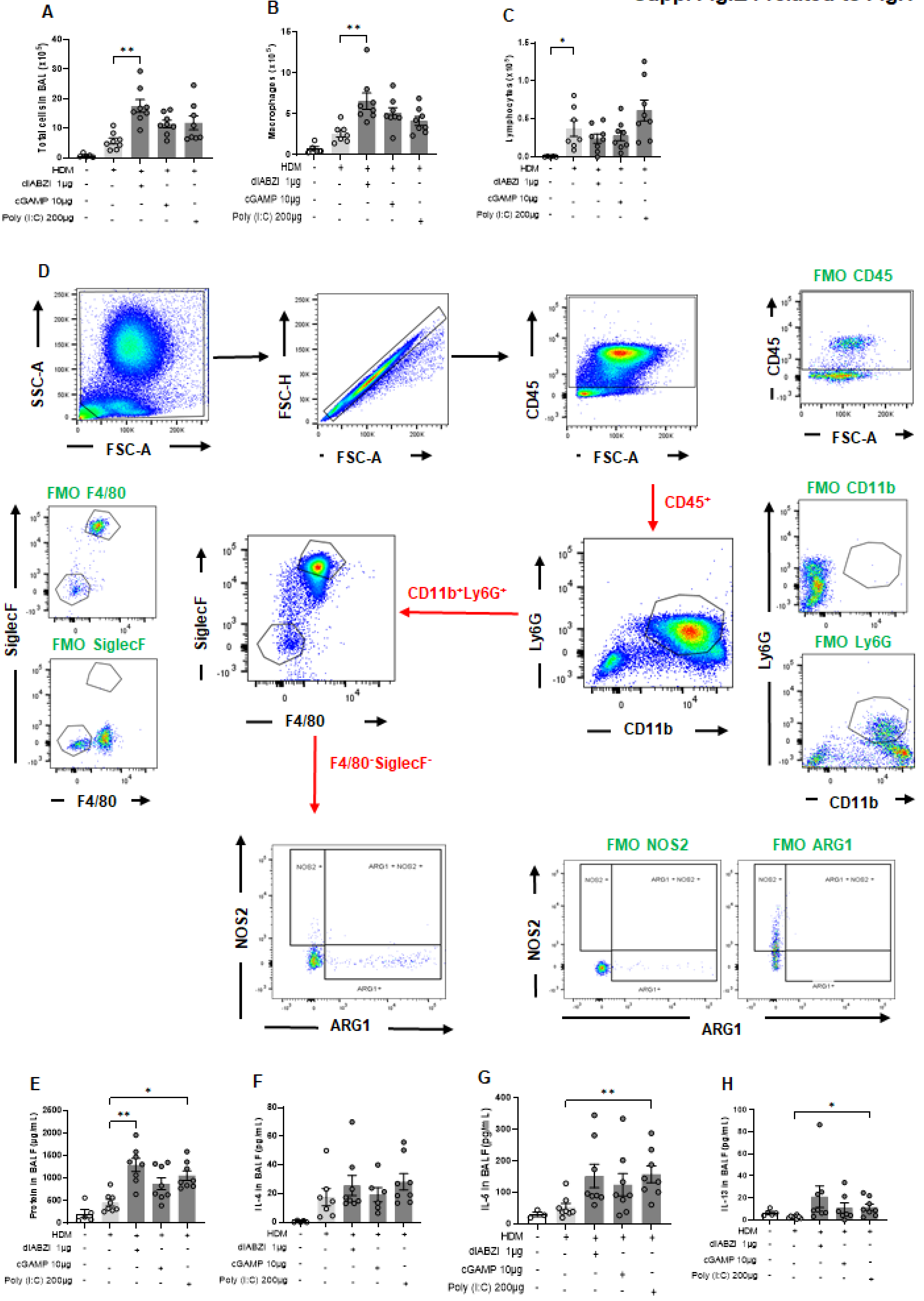

Suppl Fig.E4 related to Fig.1

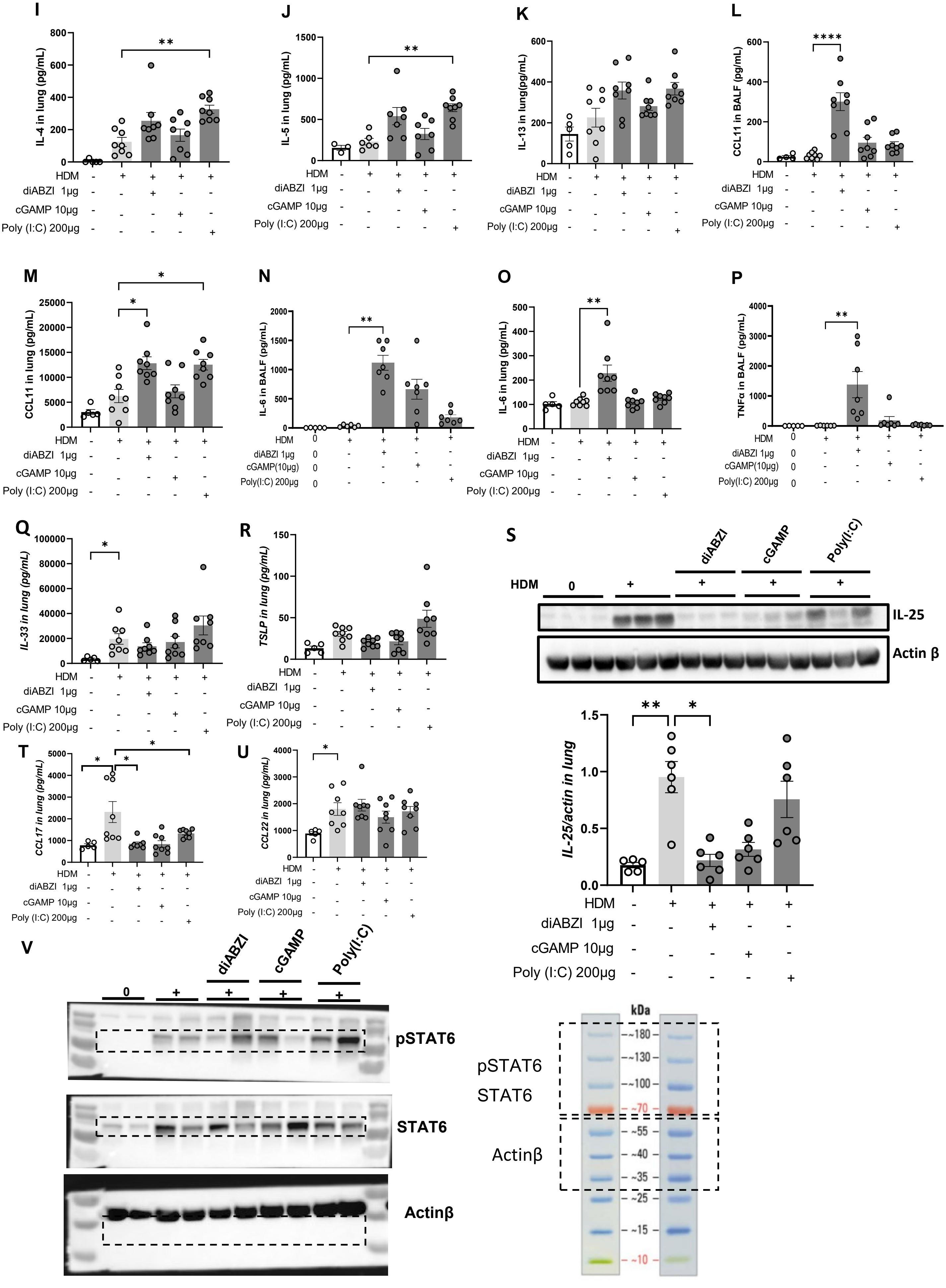

Suppl Fig.E5 related to Fig.2

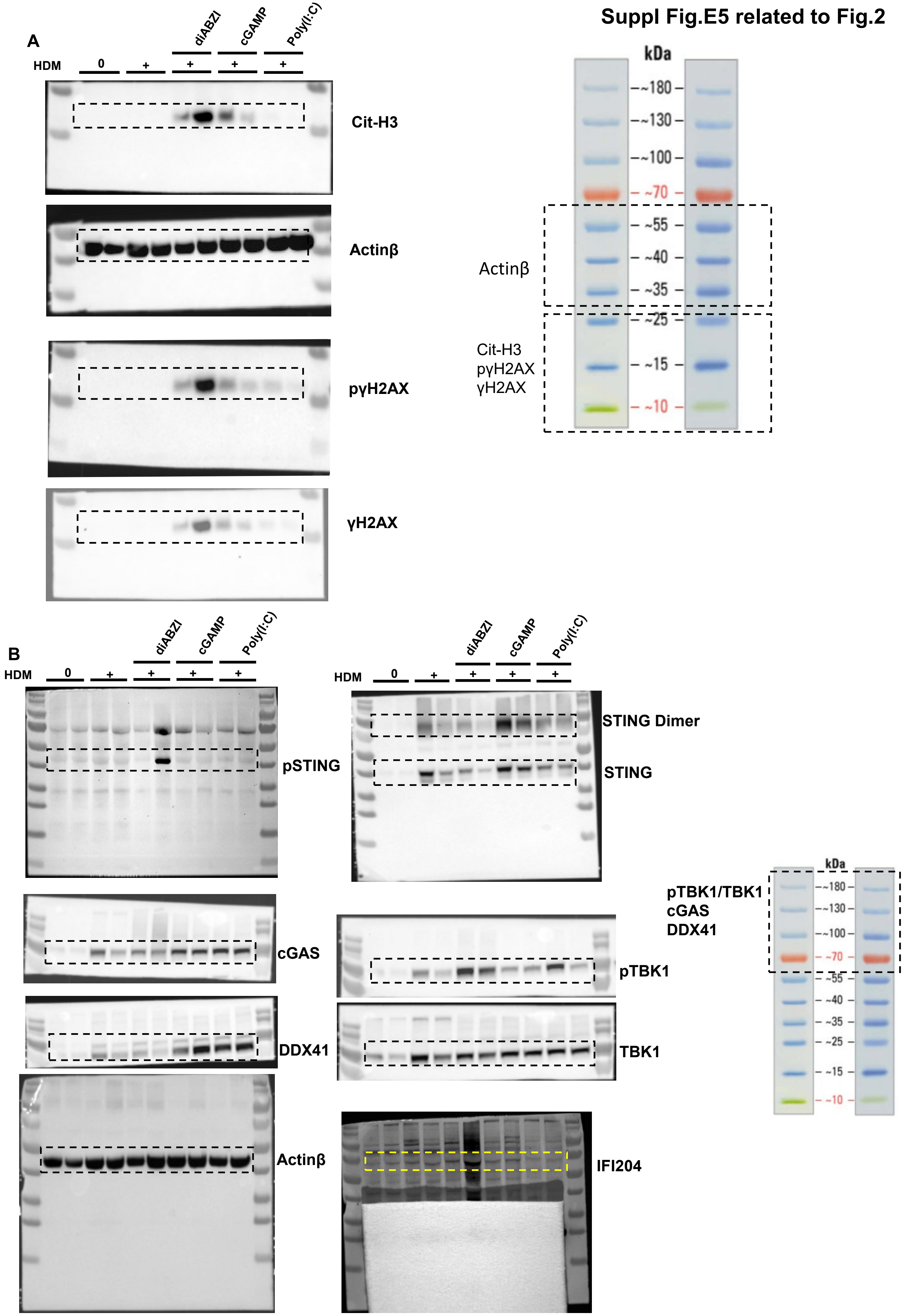

Suppl Fig. E5 related to Fig.2

C

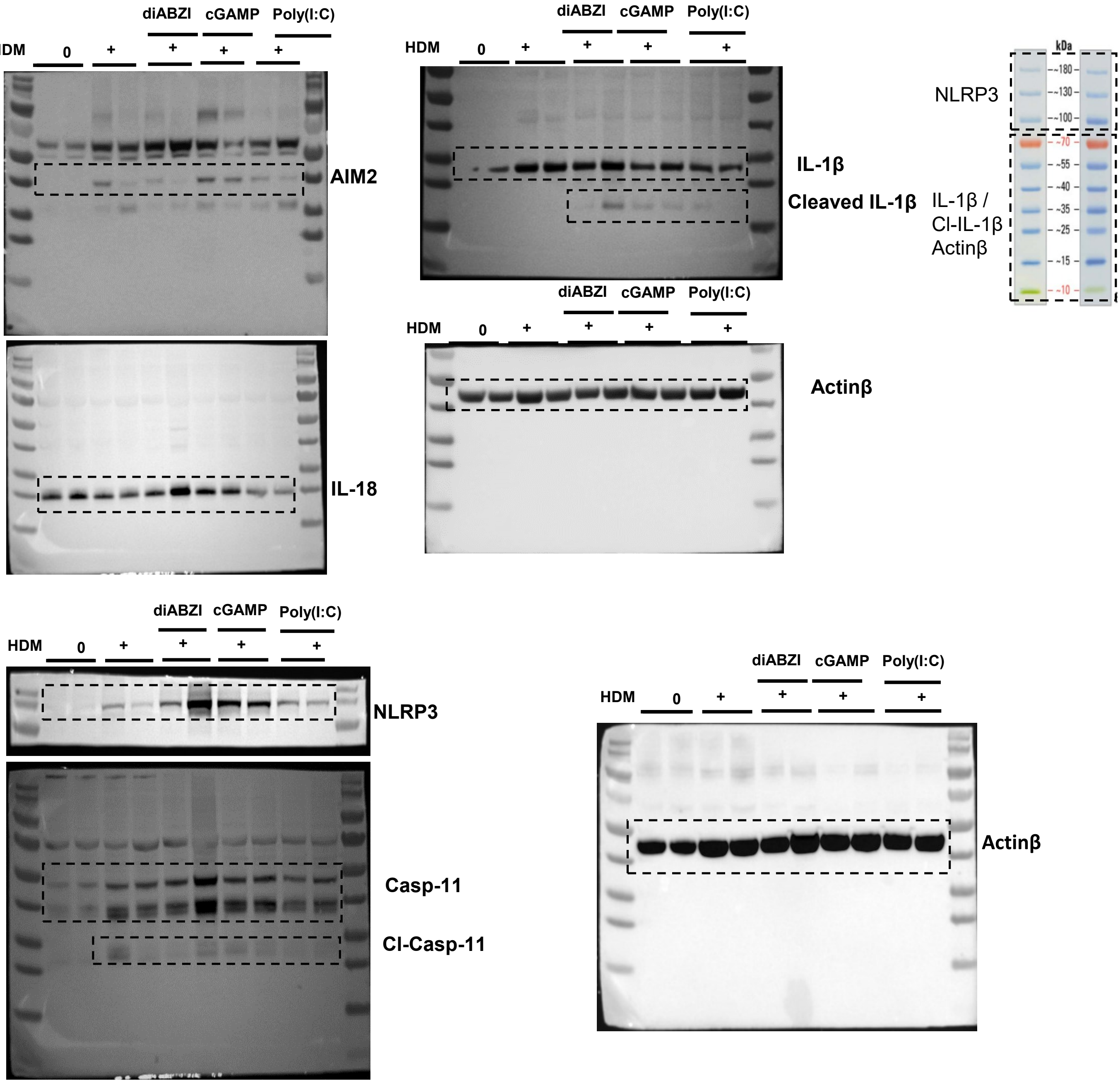

Suppl Fig. E5 related to Fig.2

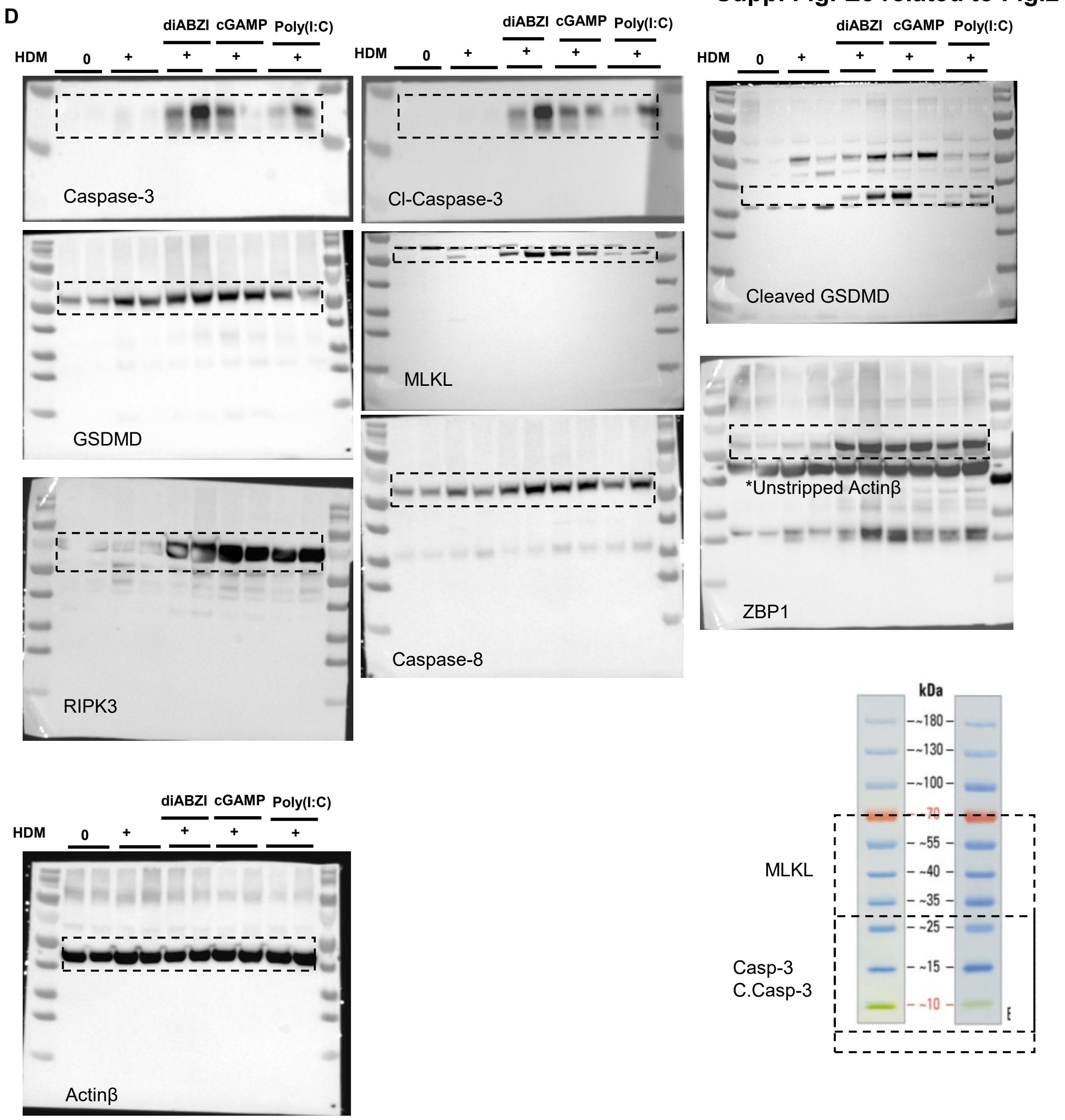

Suppl Fig. E5 related to Fig.2

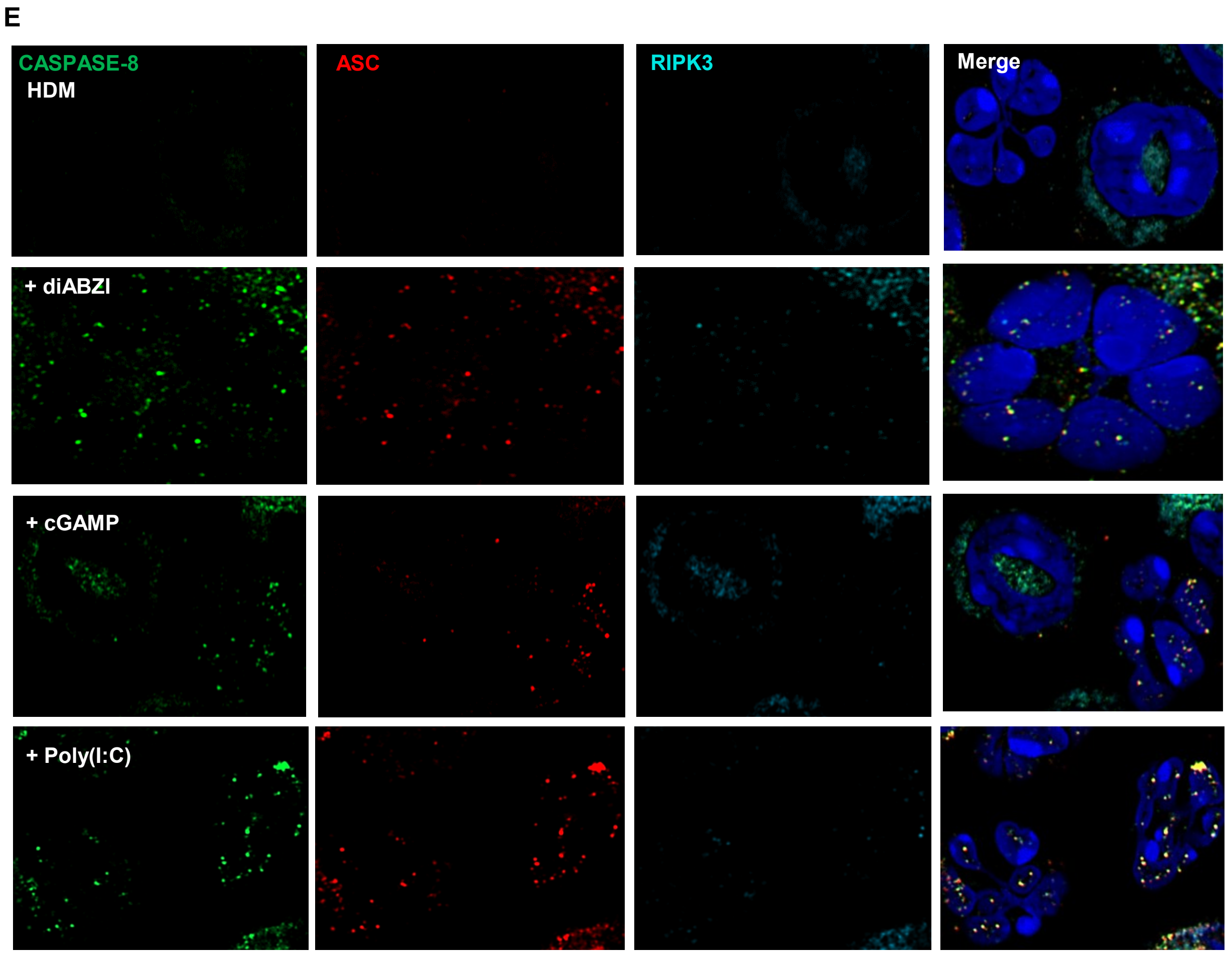

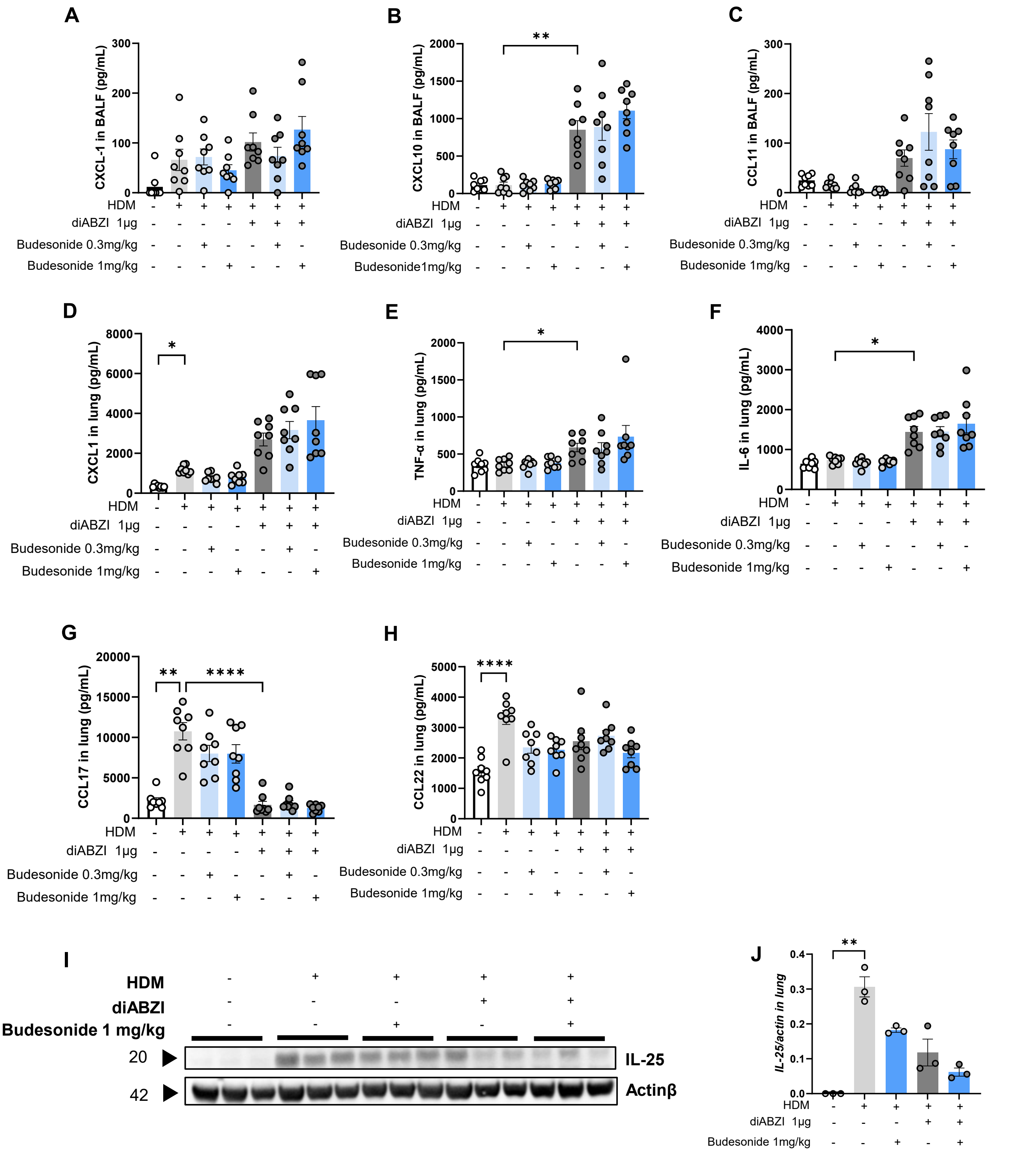
A
CXCL-1 in BALF (pg/mL)
B
CXCL10 in BALF (pg/mL)
C
CCL11 in BALF (pg/mL)
D
CXCL1 in lung (pg/mL)
E
TNF-α in lung (pg/mL)
F
IL-6 in lung (pg/mL)
G
CCL17 in lung (pg/mL)
H
CCL22 in lung (pg/mL)
HDM
diABZI 1µg
Budesonide 0.3mg/kg
Budesonide 1mg/kg
I
HDM
diABZI
Budesonide 1 mg/kg
20
42
IL-25
Actinβ
J
IL-25/actin in lung

Suppl Fig.E6 related to Fig. 3

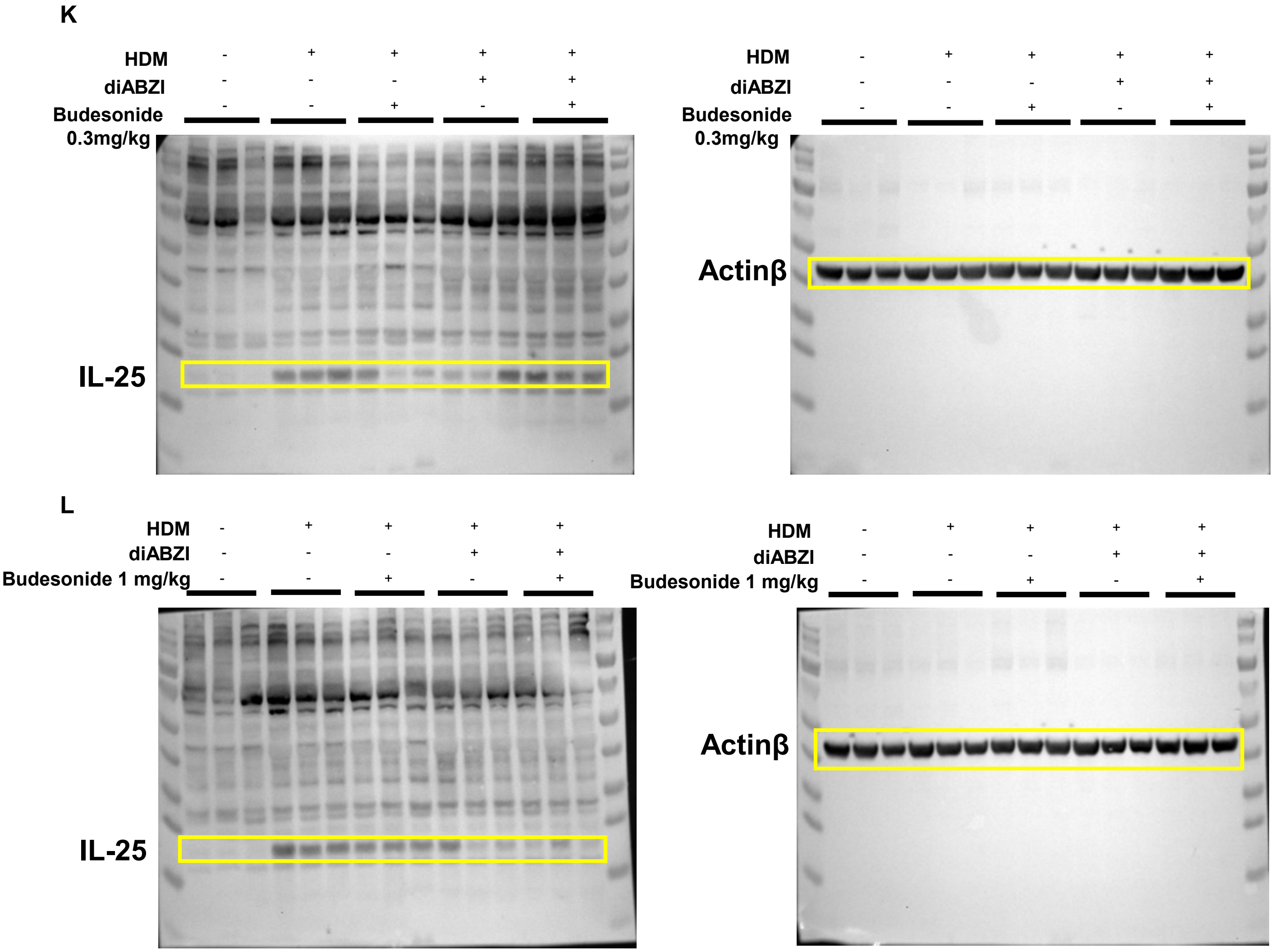

Suppl Fig. E7 related to Fig.4

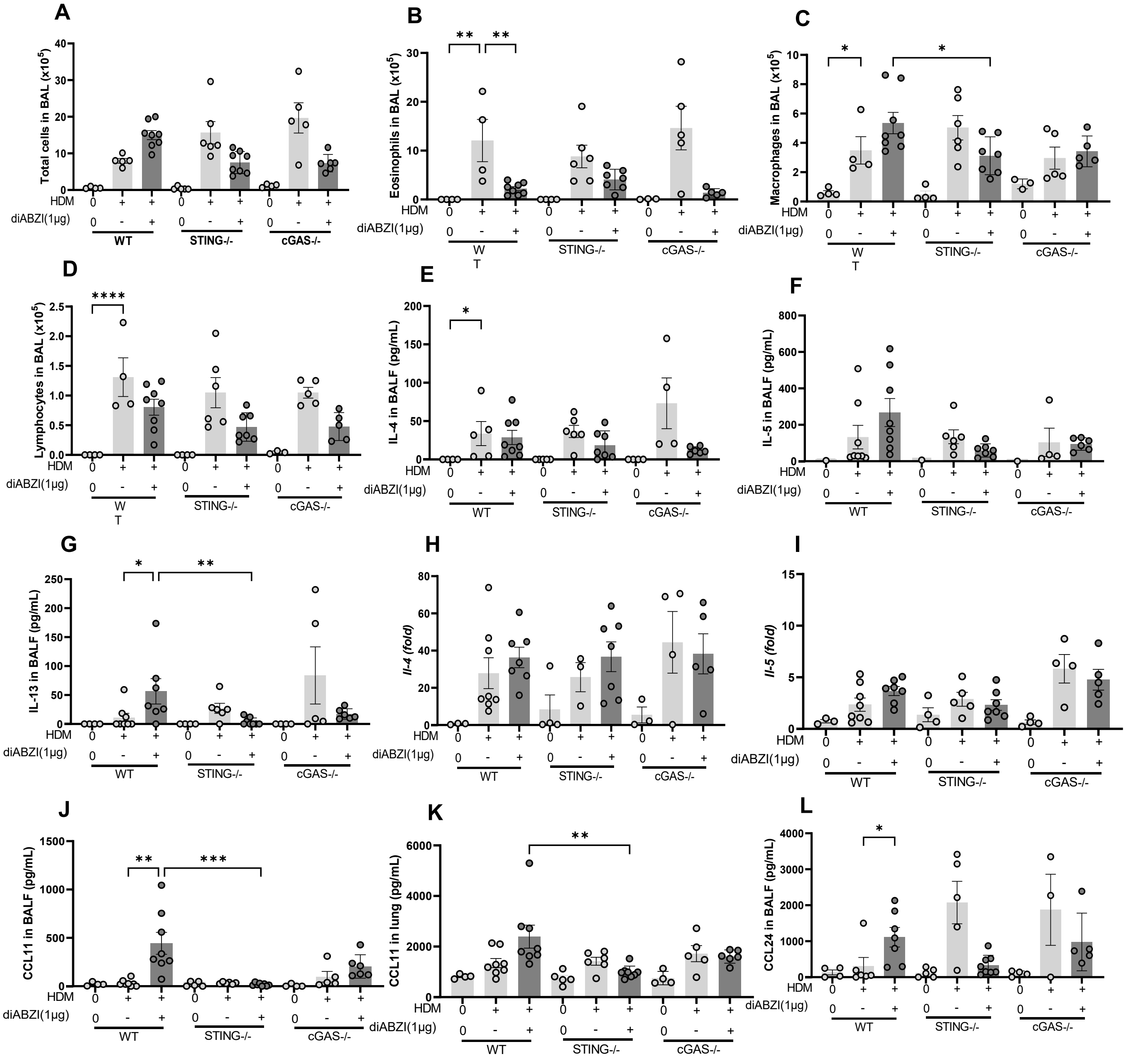

Suppl Fig. E7 related to Fig.4

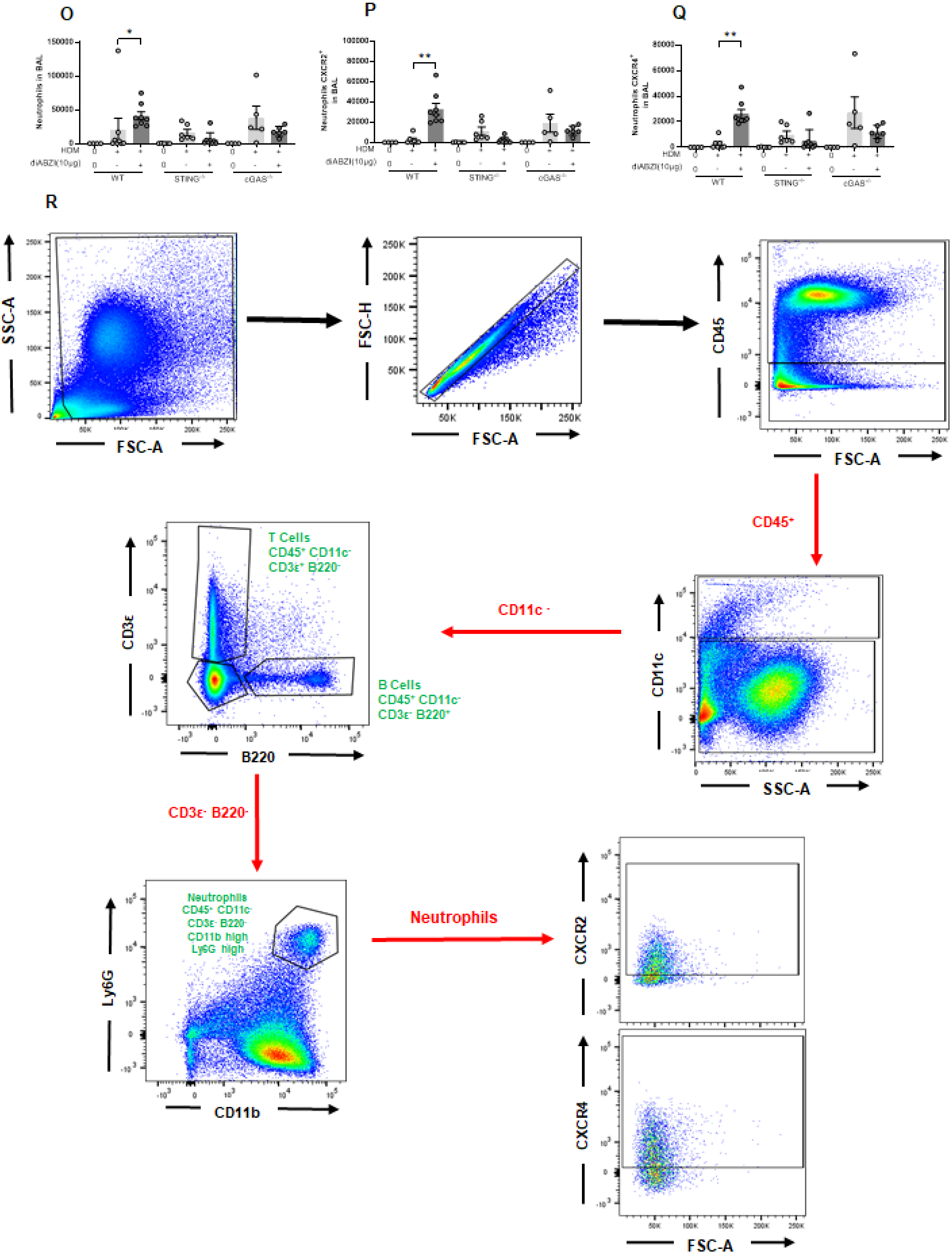

Suppl Fig.E8 related to Fig.5

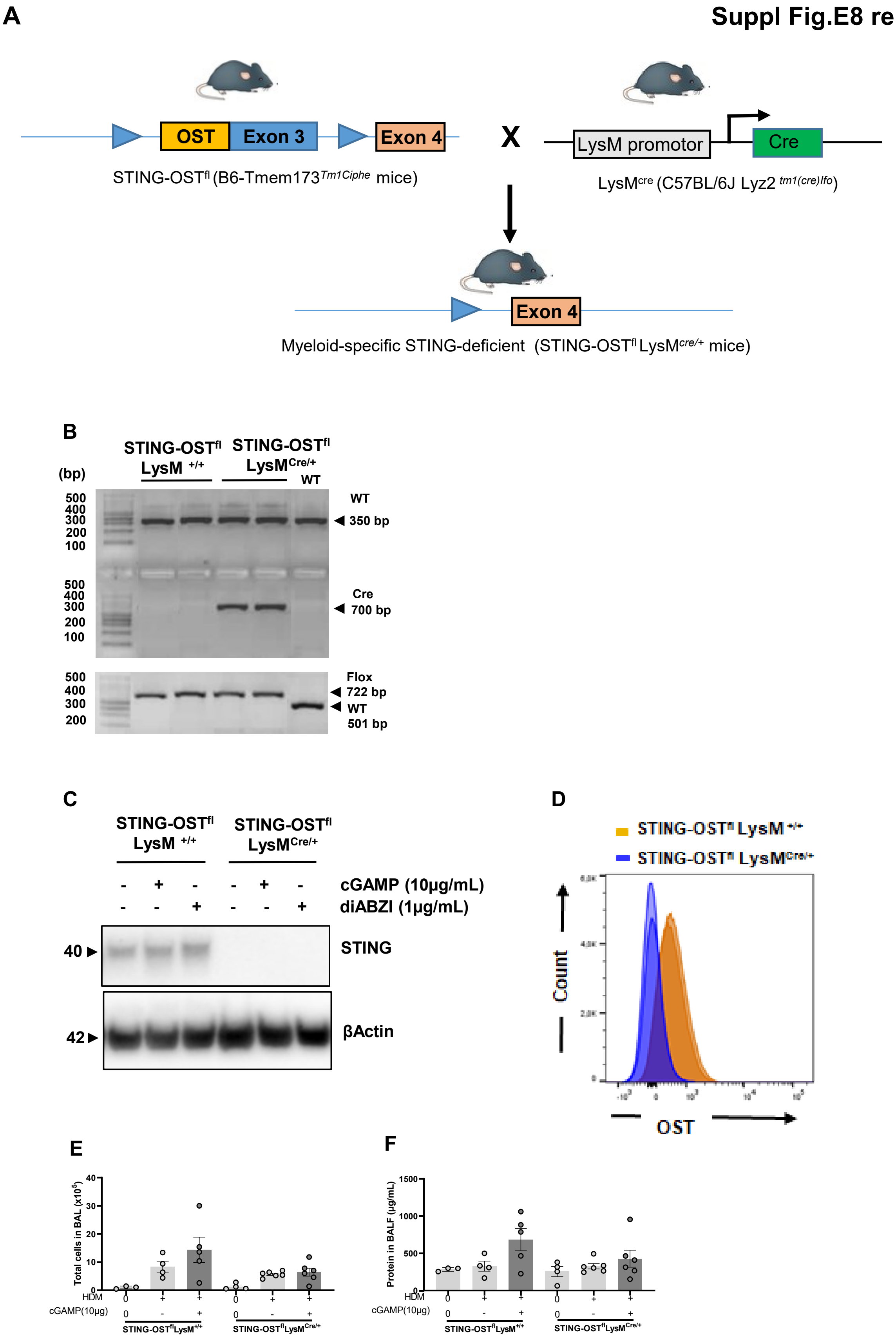

Suppl Fig.E8 related to Fig.5

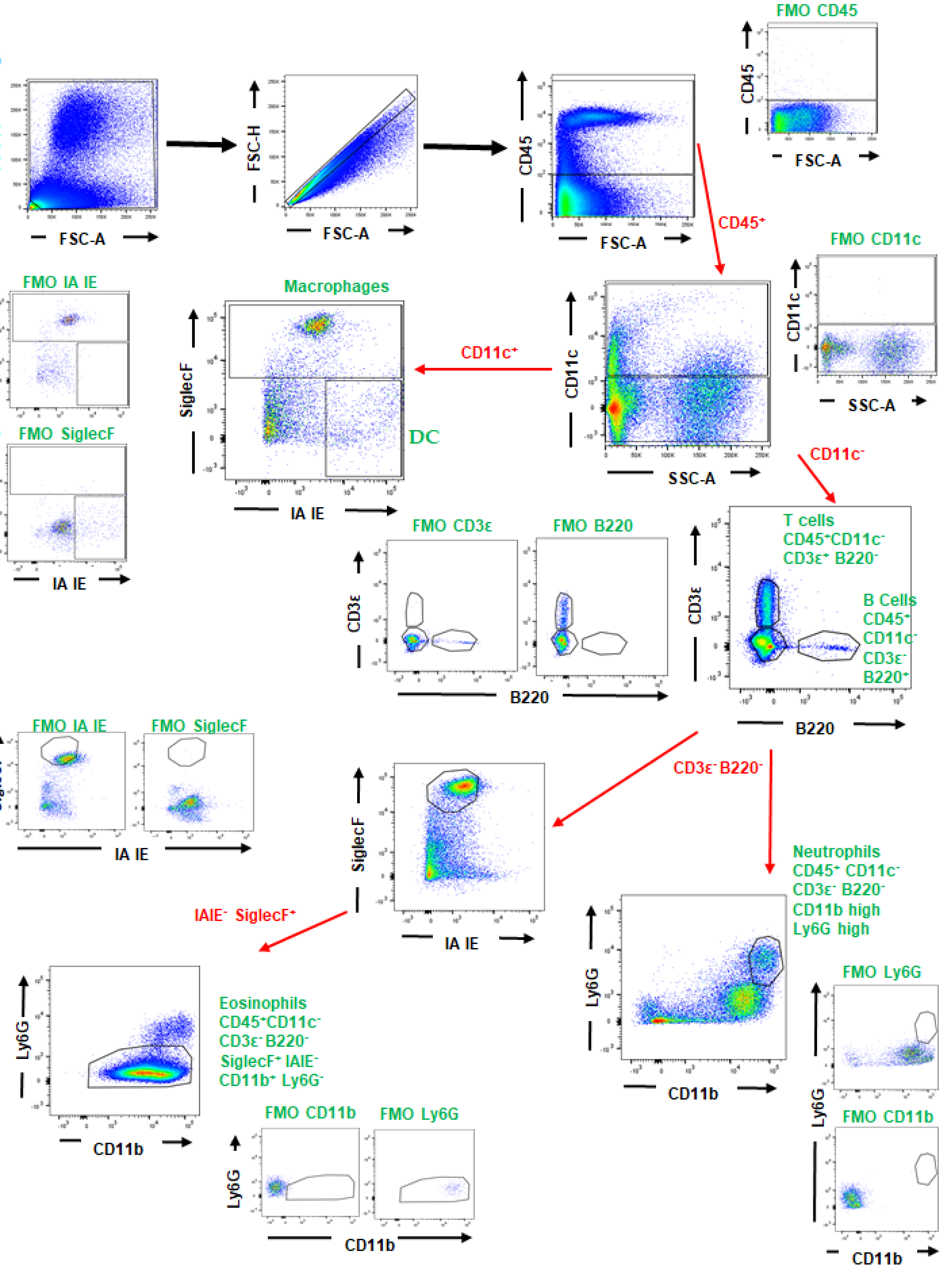

Suppl Fig.E9 related to Fig.6

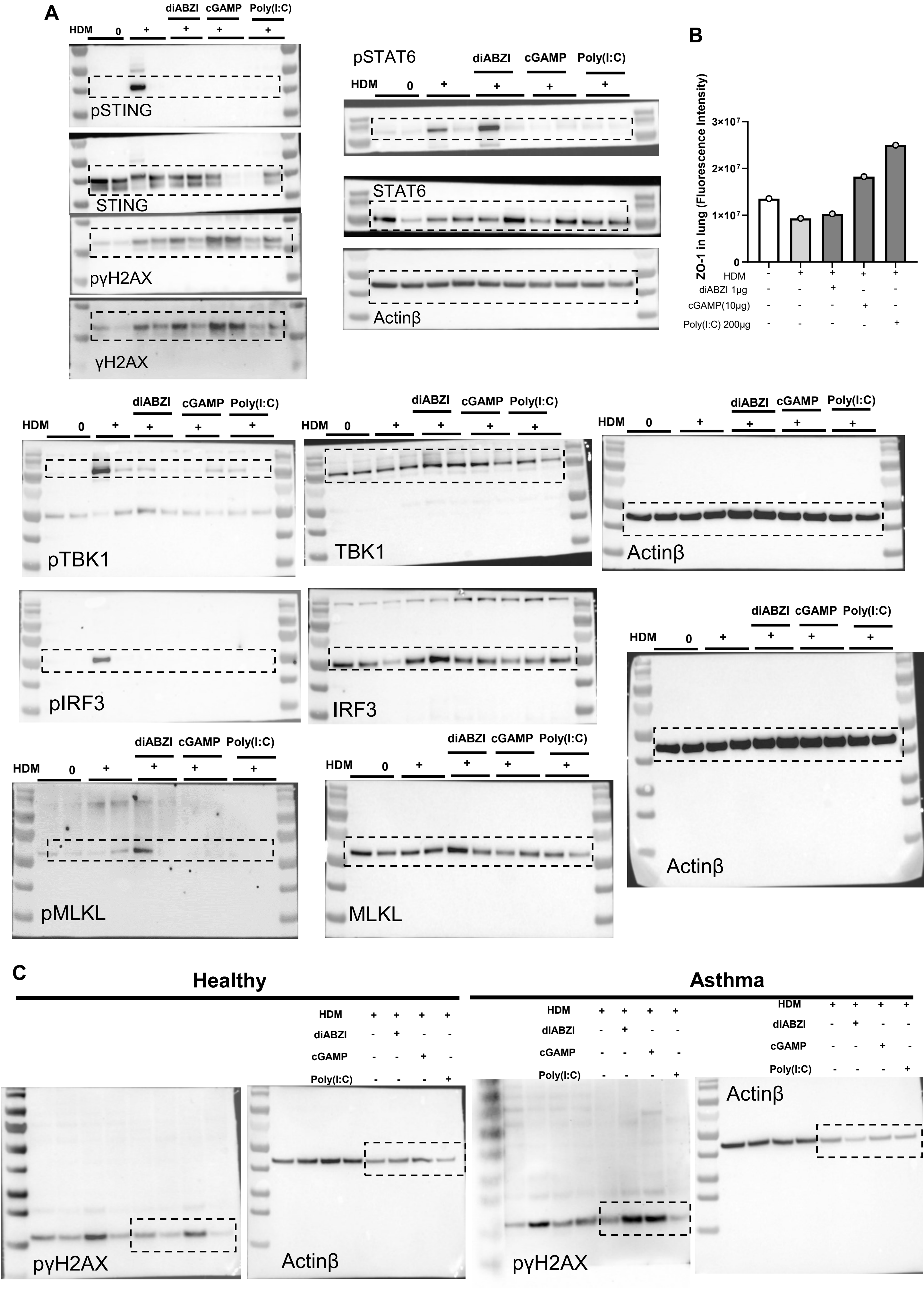

Suppl Fig. E10 related to Fig.7

A

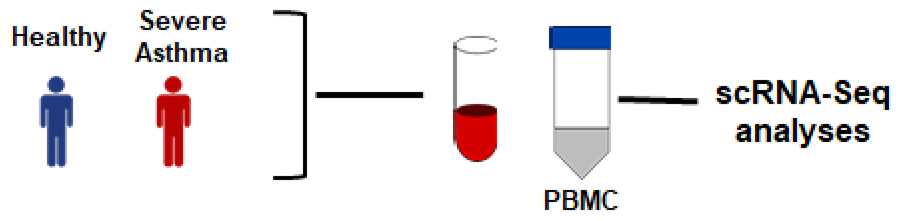

B

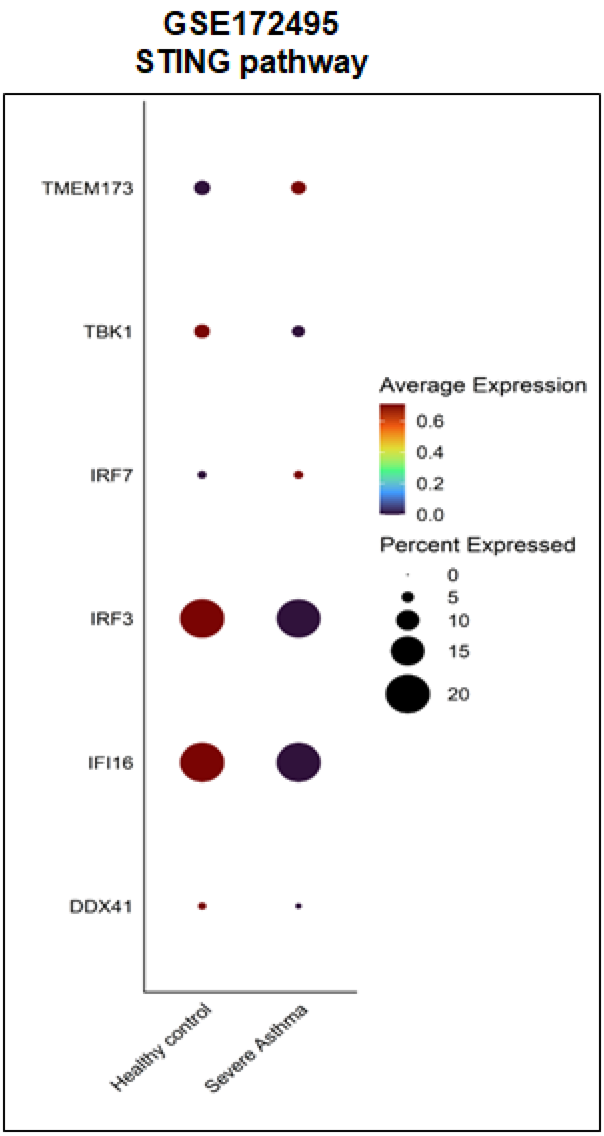

C

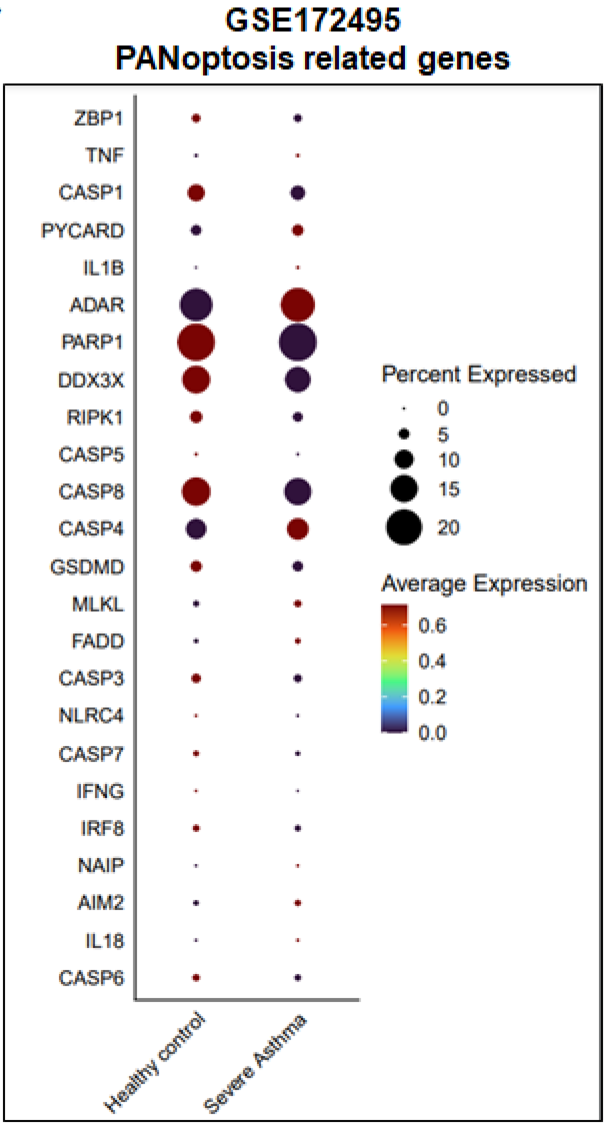

Suppl Fig. E11 related to Fig.7

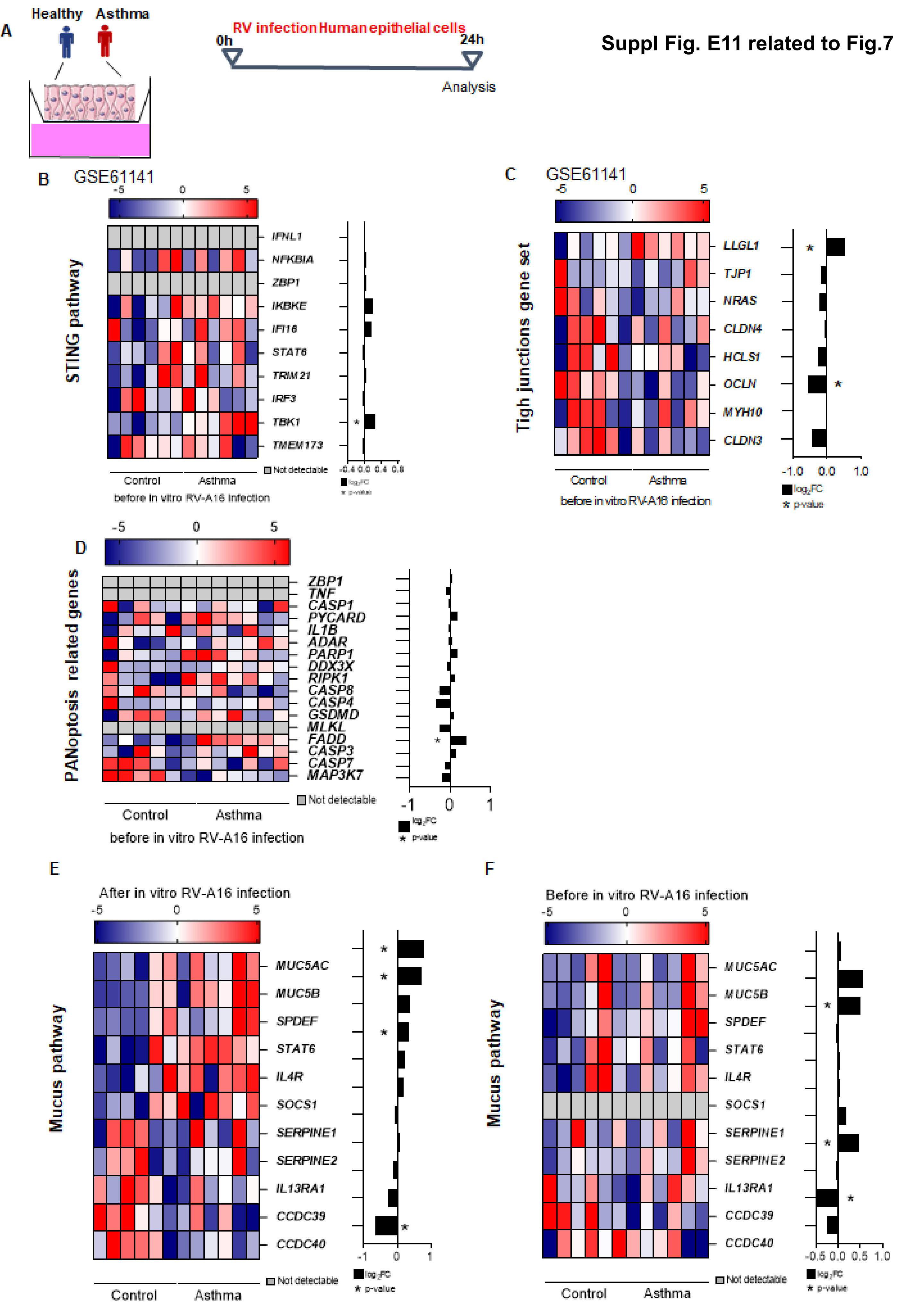

Suppl Fig. E11 related to Fig.7

G

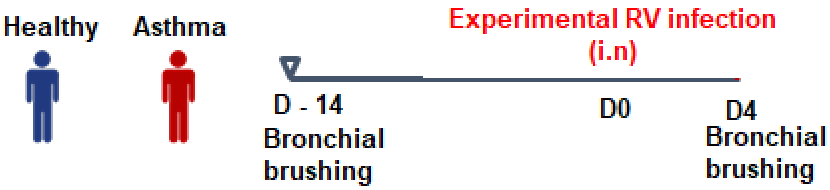

H GSE185658

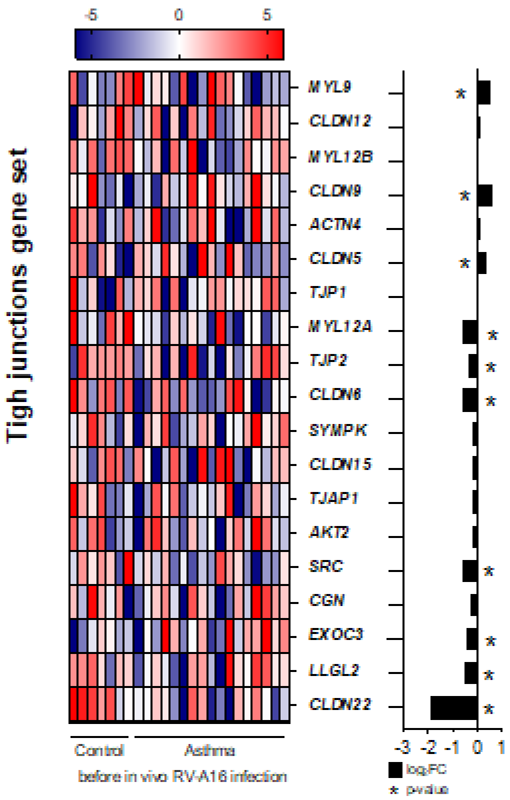

I GSE185658

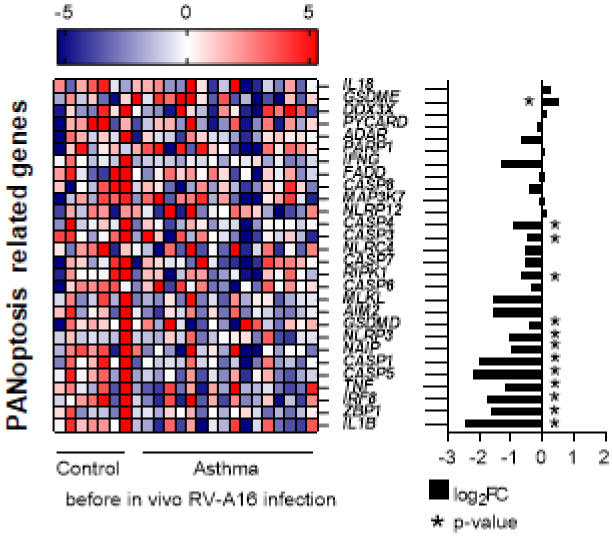

Supplement: Supplementary file 2 — Figure S1. [file ALL-80-715-s003.pdf]
